# Potential global distribution of *Aleurocanthus woglumi* considering climate change and irrigation

**DOI:** 10.1371/journal.pone.0261626

**Published:** 2021-12-20

**Authors:** Antigoni Akrivou, Iro Georgopoulou, Dimitrios P. Papachristos, Panagiotis G. Milonas, Darren J. Kriticos

**Affiliations:** 1 Scientific Directorate of Entomology and Agricultural Zoology, Benaki Phytopathological Institute, Kifissia, Attica, Greece; 2 Commonwealth Scientific and Industrial Research Organisation (CSIRO), Black Mountain Science & Innovation Park, Canberra, ACT, Australia; 3 University of Queensland, School of Biological Science, St. Lucia, QLD, Australia; USDA Forest Service, UNITED STATES

## Abstract

Citrus blackfly, *Aleurocanthus woglumi* Ashby (Hemiptera: Aleyrodidae), is an important agricultural quarantine pest, causing substantial economic losses to citrus and many other cultivated crops. *Aleurocanthus woglumi* is found in tropical and subtropical regions but is presently unknown in Europe and the Mediterranean Basin. We used CLIMEX to model the potential distribution of *A*. *woglumi* under an historical climate scenario (centred on 1995), including a spatially explicit irrigation scenario. We found that *A*. *woglumi* could potentially invade the Mediterranean Basin, and south-east Asia, including Australia. There is potential for it to invade most of sub-Saharan Africa. Irrigation is revealed as an important habitat factor affecting the potential distribution of *A*. *woglumi*, increasing its potential range by 53% in Asia. Under a future climate scenario for 2050, its potential distribution increased across all continents except Africa, where potential range expansion due to relaxation of cold stresses was limited, and was offset by range decrease due to lethal heat or dry stress. As global climates warm, Europe is likely to face a substantial increase in the area at risk of establishment by *A*. *woglumi* (almost doubling under the 2050 irrigation scenario). The biosecurity threat from *A*. *woglumi* is significant in current citrus production areas and poses a challenge to biosecurity managers and risk analysts.

## Introduction

Invasive alien species (IAS) constitute major threats to ecosystem dynamics [1–4], food security [5], and economic development [5–9]. Globalization is driving biological invasions with increasing trade [10], worldwide transport, and global travel and tourism [11]. Pest invasions have increased in recent decades [12,13], and the impacts of these pests on the environment [14], agricultural production [15] and human health [16] are profound. Consequently, appropriate phytosanitary measures are often economically justified to protect regions from accidental or intentional introduction of non-native species [17]. Pest risk maps and pest risk analyses (PRA) are fundamental tools for managing global plant biosecurity [18,19]. Pest risk maps give information regarding the potential spatial distribution of invasive species supporting strategic decisions concerning strategies to mitigate or ameliorate risks [20,21]. Climatic changes are expected to affect the distribution and abundance patterns of many species, with potential range expansions into cooler areas and contractions from warmer, drier areas [22,23]. Climate change is no longer a theoretical factor influencing pest risks, with compelling evidence of changes in pest risk attributable to recent changes in climate [24]. It is therefore prudent to consider the emerging effects of climate change when assessing pest risks and potential biosecurity policies, regulatory measures, and other actions to mitigate these risks.

The citrus blackfly, *Aleurocanthus woglumi* Ashby (Hemiptera: Aleyrodidae), is an important whitefly pest in several countries [25,26], and can infest more than 300 host plants, including cultivated plants, ornamentals and weeds of which citrus is a favoured host [26]. Direct damage by *A*. *woglumi* is caused by continuous sucking of leaf sap [27], whereas indirect damage is associated with the development of a sooty mold (*Capnodium* sp.) causing negative physiological changes to plants [28]. Physiological changes caused by these direct and indirect effects decelerate plant growth and reduce the quality, size and number of fruits [27]. In Mexico and Florida, short-term (< 1 year) infestations were found to reduce fruit production by up to 50%, and longer-term infestations frequently resulted in almost complete loss of production. In Pakistan, losses due to *A*. *woglumi* infestations have been estimated at 5 to 10% with occasional losses as high as 50 to 60% [29]. In severe infestations, yield in citriculture can be reduced by 80% [27].

Climatic variables, especially temperature and rainfall, are important factors affecting the infestation potential of *A*. *woglumi* [28]. In tropical environments, all life stages of *A*. *woglumi* may be found throughout the year, but reproduction ceases during cold periods [30]. Females favour oviposition sites in tree canopies with high humidity because the latter influences egg hatch and nymph survival [28,31]. Each female may lay more than 100 eggs in a characteristic spiral pattern on the underside of young leaves in batches (average 38) [32]. Depending on environmental conditions, the life cycle takes 2–4 months to complete, and there are up to six generations annually. Development times of different stages are as follows: egg 11–20 days; three larval instars 7–16, 5–30 and 6–20 days, respectively; pupa 16–80 days; adult lifespan probably 6–12 days [30,33].

*Aleurocanthus woglumi* was first detected in India in 1910, and in 1915 it was reported in other parts of Asia [34]. In India it is established in Amravati, Nagpur and Wardha districts of Maharastra as a pest of citrus. In the Western Hemisphere, *A*. *woglumi* was first recorded in Jamaica in 1913 and from Jamaica it spread rapidly throughout the Caribbean region into Central America (Cuba, Panama, Costa Rica, Bahamas, Haiti) [29,34]. It was also recorded in Mexico where it may have been introduced on shipments of mango cuttings from India or from boats from Central America. By 1940 it had become a serious threat to citrus in Mexico [35]. Introductions to the United States have occurred several times. *Aleurocanthus woglumi* was recorded in Florida (1934) where it is now widespread throughout the north-central and southern parts of the state, and Texas (1955), where it was eradicated but later became established [29,35,36]. In Brazil, *A*. *woglumi* was first detected in 2001, in Belém, Pará state in the north, then soon became widespread in the country [37–39]. *Aleurocanthus woglumi* was also discovered near Durban, South Africa in January 1959, and was established in and around Durban on the Natal coast [40]. It is presently unknown from Europe and the Mediterranean Basin [41]. Host plants for planting and host cut flowers or branches have been identified as the main pathways for entry into new countries [30].

Biosecurity agencies are interested to know the potential distribution of *A*. *woglumi*, as a means of understanding and better managing the pests risks it poses. EFSA used the Köppen-Geiger climate classification system to determine that *A*. *woglumi* occurs in climatic zones that also occur in European countries, making them potentially suitable for establishment [30]. A model that relies on the thermal requirements of the insect and global climate change scenarios has been used to forecast changes in the number of generations that might be expected annually in the state of Pará in Brazil [42].

CLIMEX (Hearne Scientific Software, Melbourne, Australia) is frequently used in applied ecological studies by government agencies and research organisations for a range of applications including pest risk assessment, exploring the potential effects of climate change and supporting biological control research [21,43]. CLIMEX is a hybrid inductive-deductive ecological niche model. Using CLIMEX, the factors limiting a species’ range can be inferred from its known geographical distribution, relative abundance or seasonal phenology. Where available, ecophysiological information from experimental domains can help inform the parameterisation of growth indices and factors in CLIMEX such as the base temperature for development and the onset of stresses. CLIMEX is a dynamic model with the ability to incorporate the interactions between environmental variables and functions across all latitudes, climate zones and seasons as required for biosecurity and global change applications [44]. Because its stress and growth functions are arranged in accordance with the Law of Tolerance [45,46] and the Law of the Minimum [47], CLIMEX is relatively immune from the novel climates transferability problems that challenge correlative species distribution models [48,49].

In this study we use CLIMEX to assess the climatic potential for establishment of *A*. *woglumi* in new regions, considering the geographical locations where irrigation is practiced. We apply a climate change scenario to highlight emerging areas at risk that should be factored into pest risk analyses and the formulation of biosecurity policies regarding *A*. *woglumi*.

## Materials and methods

### CLIMEX

CLIMEX is used to estimate the potential establishment of species using climate information and produces maps describing the climate suitability for population growth and survival, distinguishing between conditions for establishment or ephemeral occupancy. Here, we used CLIMEX 4.1 (Hearne Scientific Software, Melbourne, Australia) to estimate the global potential distribution of *A*. *woglumi*. The CLIMEX “Compare Locations” model uses a meteorological database consisting of monthly long-term average climatic variables for any number of locations [44]. The current distribution of a species can be used to infer its climate preferences, especially the stresses that mostly limit a species’ range. The Ecoclimatic Index (EI) represents the overall climatic suitability of a region for the modelled species. The Ecoclimatic Index (EI) combines the growth and stress indices. An EI of 0 indicates that the location is not favourable for the long-term survival of the species. A maximum EI value of 100 is only achievable under stable climatic conditions, usually found in equatorial locations. The Growth Index is derived using a Temperature Index (TI) and a Moisture Index (MI) that describe the modelled species’ response to temperature and moisture conditions and indicates the climate suitability for population growth. The weekly Growth Index (GI_W_) is integrated to give an annual Growth Index (GI_A_). The stress indices (cold, hot, dry, wet), and occasionally their interactions (hot-dry, cold-dry, hot-wet and cold-wet), represent potential limiting factors that restrict a species’ ability to survive. Stresses largely define a species’ potential geographical distribution, while the growth indices mostly describe the potential for population growth within that range boundary [44]. CLIMEX results were exported into GIS software (ArcGIS Pro, Version 2.7.1, Redlands, CA: Environmental Systems Research Institute, Inc., https://www.esri.com).

### Historical climate data

The meteorological files for projecting the potential distribution of *A*. *woglumi* contain site climate data for daily minimum temperature (Tmin), daily maximum temperature (Tmax), monthly precipitation (Rainfall), relative humidity at 9 am (RH 0900) and relative humidity at 3 pm (RH 1500). Historical meteorological data (period 1981–2010) were obtained from CliMond [50]. The CliMond climate dataset consists of gridded historical climate data and some future climate scenario data at 10’ or 30’ spatial resolution. In this study, 30’ climate data were used.

### Climate change scenario

There are substantial, irreducible uncertainties in estimating future climates. The most sensitive uncertainty appears to be the rate of emission of greenhouse gases through time [51]. The realised emission path will depend on factors such as the pattern of economic activity, geopolitical factors such as the willingness to decarbonise economies and technological and economic developments affecting the cost of renewable energy. History has underlined how these factors cannot be reliably predicted; hence, a future climate scenario that is internally consistent but relies on these uncertain factors cannot, in turn, be considered a reliable prediction of future climatic conditions. Put simply, we cannot reliably pick which emission scenario is the “correct” one, so we should reframe the problem from trying to predict the future conditions to trying to identify potential emerging problems by stress-testing the biophysical model with a climate model based on an extreme scenario.

In this study, in addition to modelling the potential distribution of *A*. *woglumi* under historic climate conditions and irrigation, we choose to use irrigation incorporated in a near-term (averaging period 2040–2059) business as usual emission scenario, Representative Concentration Pathway 8.5 (RCP8.5), realised with a popular global climate model (ACCESS1-0). The RCP8.5 pathway arises from little effort to reduce emissions and represents a failure to curb warming by 2100. It is characterized by increasing greenhouse gas emissions over time and represents scenarios in the literature leading to high greenhouse gas concentration levels [52]. As suggested above, we do not use the model to try to predict the future potential distribution of *A*. *woglumi*, but rather to understand the direction of range changes and the relative sensitivity of the potential distribution to expected future climatic changes. In essence, we are undertaking a sensitivity analysis of our CLIMEX model results to expected climate changes. This allows us to comment on what invasion threats are likely to emerge or wane in the near to medium term future. Hence, the model results should not be considered a prediction of what will come to pass. Rather, they are intended to be used as a basis for stress-testing biosecurity policies.

### Location records

The historical distribution of *A*. *woglumi* used to build the CLIMEX model was assembled mostly from published studies and databases such as EPPO-Q-bank, and the American Museum of Natural History (S1 Table). Province/region-level occurrence records of *A*. *woglumi* were retrieved also from the literature and represent administrative regions where the pest has been reported, limiting their value for precise model calibration. Fuzzy logic is required to compare these records to the model results. The provincial/regional records are in S1B Table, while the point locations (cities, town, municipalities) are presented in S1A Table. The coordinates listed in S1B Table represent the centroid of each polygon. The native range data was mostly used for model-fitting, with data elsewhere used for model validation. In total, the dataset consisted of 179 locations.

### Model parameterisation

The fitted parameter values for *A*. *woglumi* are given in Table 1, with an explanation of each parameter.

**Table 1:** CLIMEX model parameter values for *Aleurocanthus woglumi*.

Parameter	Description	Value
**Moisture**		
SM0	lower soil moisture threshold	0.1^a^
SM1	lower optimum soil moisture	0.3 ^a^
SM2	upper optimum soil moisture	1.2 ^a^
SM3	upper soil moisture threshold	2 ^a^
**Temperature**		
DV0	lower threshold	14°C
DV1	lower optimum temperature	26°C
DV2	upper optimum temperature	32°C
DV3	upper threshold	43°C
**Cold stress**		
TTCS	cold stress temperature threshold	0°C
THCS	temperature threshold stress accumulation rate	-0.01 week^-1^
DTCS	cold stress degree-day threshold	6°C days
DHCS	degree-day cold stress accumulation rate	-0.001 week^-1^
**Heat stress**		
TTHS	heat stress temperature threshold	43°C
THHS	temperature threshold stress accumulation rate	0.01 week^-1^
**Dry stress**		
SMDS	soil moisture dry stress threshold	0.1 ^a^
HDS	dry stress accumulation rate	-0.05 week^-1^
**Threshold Heat Sum**		
PDD	number of degree-days above DV0 needed to complete one generation	985°C days
**Irrigation scenario**	4.5 mm day^-1^, applied as top-up	

^a^Expressed as a proportion of soil moisture holding capacity, where 0 indicates oven dry and 1 the field capacity (saturation).

#### Stress indices

*Cold stress*. There appear to be two cold stress mechanisms limiting the distribution of *A*. *woglumi*: a lethal temperature sensitivity and a degree day energetic balance mechanism. *Aleurocanthus woglumi* does not survive exposure to temperatures below freezing [41] and there is no known physiological mechanism to allow overwintering of *A*. *woglumi* in cold climates. Accordingly, the lethal temperature cold stress threshold (TTCS) was set at 0°C (average weekly minimum temperature < 0°C) and the stress accumulation rate (THCS) at -0.01 week^-1^ (stress accumulates moderately quickly). The cold stress temperature threshold of 0°C allows persistence in high altitude areas of Fars Province in Iran, which is consistent with the current distribution records, and limits the distribution range in northern areas where *A*. *woglumi* is not known to occur. The cold stress degree day threshold was set to 6°C days per week, and the corresponding stress accumulation rate was set to -0.001 week^-1^. Under this mechanism, cold stress accumulates if there were fewer than 6 degree-days above DV0 (14°C) and provided an appropriate fit to the observed distribution in North America, South Africa, and Asia.

*Heat stress*. Heat stress parameters were set to allow persistence at all known distribution locations. While most of the native range in south-east Asia does not get above 35°C, temperatures often exceed 44°C in the Middle East (including Khuzestan province in Iran where there is a persistent population of *A*. *woglumi*) [29,53,54]. We set the heat stress temperature threshold (TTHS) to 43°C and the heat stress accumulation rate (THHS) to 0.01 week^-1^ (stress accumulates moderately quickly). The chosen threshold also accords with observations by Cherry [55] that the lethal temperatures for *A*. *woglumi* occurred between 40 and 45°C.

*Dry stress*. Soil moisture dry stress threshold (SMDS) was fitted by using the lower soil moisture growth threshold (SM0) because dry stress begins at the same soil moisture level at which development stops and adjusting the rate (HDS) to -0.05 week^-1^ to limit the distribution to tropical and subtropical regions where it has been reported. In the Middle East and India, where there are location records, agriculture is only possible because of irrigation.

*Wet stress*. Wet stress was not used to limit the range of *A*. *woglumi* in this study because it favours a humid environment and is not limited by this stress. *Aleurocanthus woglumi* has been found in Madhya Pradesh, India where annual rainfall is close to 1 372 mm, Oyo State, Nigeria with an annual rainfall over 1 000 mm, Trinidad & Tobago with an annual rainfall exceeding 1 600 mm as well as Pará and Maranhão States in Brazil with an annual rainfall over 1 065 mm according to CLIMEX meteorological data (period 1981–2010).

#### Growth Index (GI)

*Temperature Index (TI)*. The Temperature Index in CLIMEX describes the species’ response to temperature and consists of four parameters: DV0, DV1, DV2 and DV3. DV0 and DV3 are the lower and upper temperature thresholds, respectively, whereas DV1 and DV2 are the lower and upper optimum temperatures, respectively, for population growth [43]. No development occurs below 13.7°C [56] and therefore we set the value of DV0 at 14°C. Laboratory experiments found that the optimal temperature for development is 25±3°C [42], relative humidity 70–80% [41], and survivorship was greatest at 25.6°C [56]. The range of optimum temperatures was set to 26°C and 32°C for DV1 and DV2, respectively. *Aleurocanthus woglumi* does not occur in areas where temperatures exceed 43°C [30]. Moreover, Cherry [55] estimated that high temperature exposure at 45°C for 3 hours resulted in 95% adult mortality. The maximum temperature for development is usually reasonably close to the maximum temperature for survival. This is apparent in physiologically based development functions, such as models of Stinner et al. [57] and Briere et al. [58]. *Aleurocanthus woglumi* persists in areas such as the Sindh and Punjab provinces in Pakistan and the Maharastra state in India, where average long-term monthly maximum temperatures can exceed 42 ˚C (e.g., Sindh 42.9 ˚C). These lines of evidence make it reasonable to set the maximum value for development (DV3) at 43°C.

*Moisture Index (MI)*. In CLIMEX the effects of rainfall and humidity are combined into a single soil Moisture Index (MI), using a hydrological model to represent moisture availability [59]. The lower soil moisture limit for development (SM0) was set to 0.1 to indicate the permanent wilting point of the insect’s host plants, which corresponds to approximately 10% of the soil moisture level [44]. The lower optimal soil moisture (SM1) was set at 0.3, which indicates soil moisture content is 30% of available capacity, and the upper optimal soil moisture (SM2) was set at 1.2. The upper soil moisture limit for development, SM3, was set to 2.

#### Degree-days per generation (PDD) and model validation

PDD denotes the number of degree-days above DV0 required to complete a single generation. It acts as a limiting condition when a minimum of one generation must be completed each year for the species to persist at a location [44]. Following Dowell and Fitzpatrick [56], the heat sum above a 13.7°C developmental threshold to complete one generation of *A*. *woglumi* was 981 ± 10°C degree-days. Dowell and Steinberg [60] reported the sum of degree-day above DV0 for *A*. *woglumi* at 985 ± 22, 990 ± 18 and 982 ± 20°C days, depending on nitrogen fertilizer. We set PDD to 985°C days.

Among various methods used for model validation, most CLIMEX validation exercises focus on comparing the modelled potential distributions with the known occurrences in geographical areas not used for parameter fitting in pests’ native ranges [61,62]. In our study, we also used observations about voltinism of *A*. *woglumi* in three known locations taken from the literature (south Florida in USA, Kuala Lumpur in Malaysia, and Panama Canal Zone) and compared them with the modelled establishment results for the pest in these locations [63].

### Irrigation scenario

Non-climatic habitat factors can have a substantial impact on cultivation and consequently on many pest species’ distributions. Irrigation is one such factor that affects soil moisture. *Aleurocanthus woglumi* has location records in the Middle East and India where agriculture is only possible because of irrigation. In the CLIMEX model under current climate conditions (natural rainfall scenario), using biologically plausible soil moisture parameters, many of these locations would be modelled as unsuitable due to drought stress. To account for the effects of irrigation, an irrigation scenario of 4.5 mm day^-1^ was applied globally as a top-up, using a composite map developed from a global map of irrigated areas [64]. The global map of irrigated areas (GMIA) indicates the irrigated areas within each 5’ grid cell. For this study, the GMIA dataset was aggregated to 30’ grid cells. A threshold of 10 ha of irrigated area was applied; for each 30’ cell, if the irrigated area was greater than or equal to 10 ha it was considered to be irrigated, otherwise it was classified as natural rainfall. A composite risk map was developed using a method that is described in detail in Yonow et al. [59]. In short, for cells classified as irrigation, the greater EI value under the irrigation or natural rainfall scenario was used. For the remaining cells, the EI value of the natural rainfall scenario was used.

### Parameter sensitivity and model uncertainty

Parametric sensitivity is the strength of the effect of each parameter on selected state variables. CLIMEX Version 4 provides tools to automate analyses. In the sensitivity analysis, the model is run for a scenario where each parameter is adjusted upward and then downward by the relevant perturbation value. In CLIMEX the perturbation ranges are set as follows: for temperature +/− 1°C, soil moisture +/− 10%, rate parameters +/− 10%, degree day temperature thresholds +/− 1°C and degree day sum (PDD) +/− 20°C days [44]. A table of all the parameters is produced, indicating the amount by which each has been adjusted and the effect this adjustment had on each of the state variables.

Model uncertainty is an estimate of incertitude in the results of the model. The automated uncertainty analysis provided by CLIMEX Version 4 uses a Latin hypercube framework to efficiently sample parameter uncertainty. The analysis produces a map indicating the proportional model agreement (%) for climate suitability. For each of the sensitivity and uncertainty analyses, the default model parameters were run for the entire world using the CM30 1995H V2 dataset.

## Results and discussion

### Current climate projections and model validation

**Fig 1:**
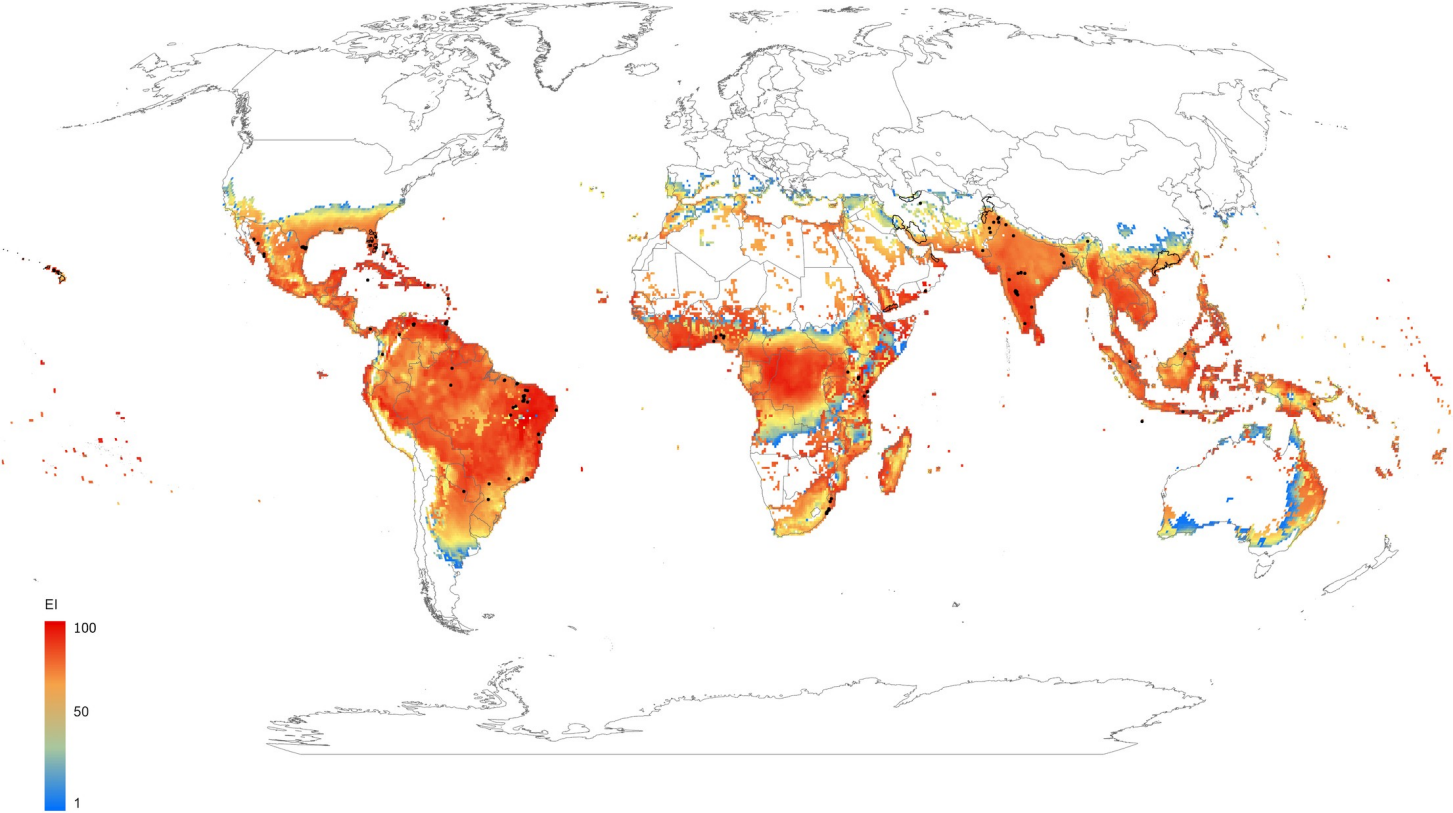
Modelled global climate suitability for *Aleurocanthus woglumi* under a historical climate scenario, represented by a composite of natural rainfall and irrigation scenarios based on irrigation areas identified by Siebert et al. [64]. The Ecoclimatic Index (EI) describes the overall climate suitability for population persistence, where 0 is unsuitable and 100 is year-round optimal conditions. Black dots indicate location records for *A*. *woglumi* (S1A Table) and black polygons the administrative areas where *A*. *woglumi* has been reported (S1B Table). This map was produced by the authors using ArcGIS Pro 2.7.1 software (@esri.com; no copyrighted material was used). Global irrigation areas [64] are used herein under a CC BY 4.0 license, with permission from Stefan Siebert, original copyright 2013. Boundary data for the countries of the world come from Natural Earth (@naturalearthdata.com; public domain) [65], and provincial/regional boundary data from geoBoundaries (@geoboundaries.org) [66]. GeoBoundaries are used herein under a CC BY 4.0 license, with permission from Daniel Runfola, original copyright 2020.

The potential distribution of *A*. *woglumi* under historical climatic conditions is presented in Fig 1. Globally, around 42 and 51 million km^2^ of land are modelled as climatically suitable for *A*. *woglumi* in the absence and presence of irrigation under current climate, respectively (Table 2). Under current climate conditions, *A*. *woglumi* is well distributed in tropical and subtropical regions throughout Asia and the Pacific, to central and southern Africa as well as parts of North and South America. The Sahara Desert, central Australia, New Zealand, Korea, most of Europe, Canada and Russia are among the countries or regions with unfavourable conditions. In terms of the model parameters, dry stress and hot stress mostly limit its distribution in desert areas and inland Australia, whereas temperature and cold stress limit its distribution in northern Europe, Canada and Russia. Climatic conditions are projected to be marginal in many warm temperate areas, such as southern Europe and northern New Zealand.

**Table 2:** Projected land area (millions km^2^) with EI>0 for *Aleurocanthus woglumi* under the natural rainfall (under current climate), irrigation (natural rainfall topped up to 4.5 mm day^-1^ on irrigable land under current climate), and climate change (using the RCP8.5 ACCESS 1–0 model) scenarios. Percent changes caused by irrigation and climate change relative to the natural rainfall and the irrigation scenarios are also shown.

	Land area projected with EI>0				Change in areas projected with EI>0		
Continent	Natural Rainfall Scenario	Irrigation Scenario	Climate Change (Natural Rainfall Scenario)	Climate Change (Irrigation Scenario)	Irrigation Change	Climate Change (Natural Rainfall Scenario)	Climate Change (Irrigation Scenario)
	million km^2^				%		
Africa	12.84	16.40	12.10	16.09	27.73	-5.76	-1.89
Asia	7.88	12.03	9.16	13.31	52.66	16.24	10.64
Europe	0.27	0.31	0.57	0.59	14.81	111.11	90.32
North America	3.50	4.38	3.77	5.02	25.14	7.71	14.61
South America	14.84	15.18	14.72	15.35	2.29	-0.81	1.12
Oceania	2.95	2.93	2.70	3.00	-0.68	-8.47	2.39

Data source: Global irrigation areas [64] are used herein under a CC BY 4.0 license, with permission from Stefan Siebert, original copyright 2013. Boundary data for the countries of the world come from Natural Earth (@naturalearthdata.com; public domain) [65].

Under irrigation, favourable habitat suitability is projected for *A*. *woglumi* in areas in which it is not known to occur or persist, such as Jordan, Madagascar, southern Japan, eastern Australia, north-eastern Argentina, north-eastern Bolivia, eastern Mozambique, areas in Somalia, North Africa and eastern Saudi Arabia (Fig 1). Distribution records for *A*. *woglumi* in Iran, India, Oman, Yemen and some locations in Sinaloa in Mexico are modelled as being climatically unsuitable under the natural rainfall scenario, but climatically suitable under the irrigation scenario (S1 Fig). Under the irrigation scenario (under current climate), the percent area of potential distribution (EI>0) is increased on all continents except for Oceania (Table 2).

Further validation was carried out by comparing the number of generations modeled and reported in the literature at various locations. Hoelmer and Grace [29] reported that in south Florida (USA) three to four generations occur annually. Our model calculated about 3.8 in the southern parts. Clausen and Berry [67] stated that close observations upon this species in Kuala Lumpur (Malaysia) produced five generations each year, which is consistent with our modelled estimate of 4.5 annual generations. In the Panama Canal Zone, at least on the Pacific side, during the dry season at least one generation is lost, making the maximum number of generations five instead of six [33]. Our model simulations estimated that *A*. *woglumi* has about 4.8 generations in the Canal Zone.

### Sensitivity analysis and model uncertainty

Parametric sensitivity analysis identifies those parameters that are most important for model accuracy. Of concern are those parameters that are both sensitive and least reliably estimated. In relation to the modelled potential range, the dry stress threshold (SMDS) is the most sensitive parameter (5.3%) (S2 Table). The remaining parameters each have less than 1% sensitivity. The dry stress threshold is well anchored in biological theory, as the permanent wilting point of plants is a well-studied and consistent phenomenon, so we can be confident in this parameter. The dry stress accumulation rate is a fitted parameter, with moderate uncertainty, though the sensitivity is also very low (0.9%). The low sensitivity in the range change in the sensitivity analysis provides confidence in the model [44]. Model uncertainty is presented in S2 Fig. The strong concordance between the areas of high model agreement in S2 Fig (red areas) and the extent of area modelled with EI>0 in Fig 1 indicate that despite the limited amount of distribution data we can be confident in the fitted parameter set.

### Future climate projections

**Fig 2:**
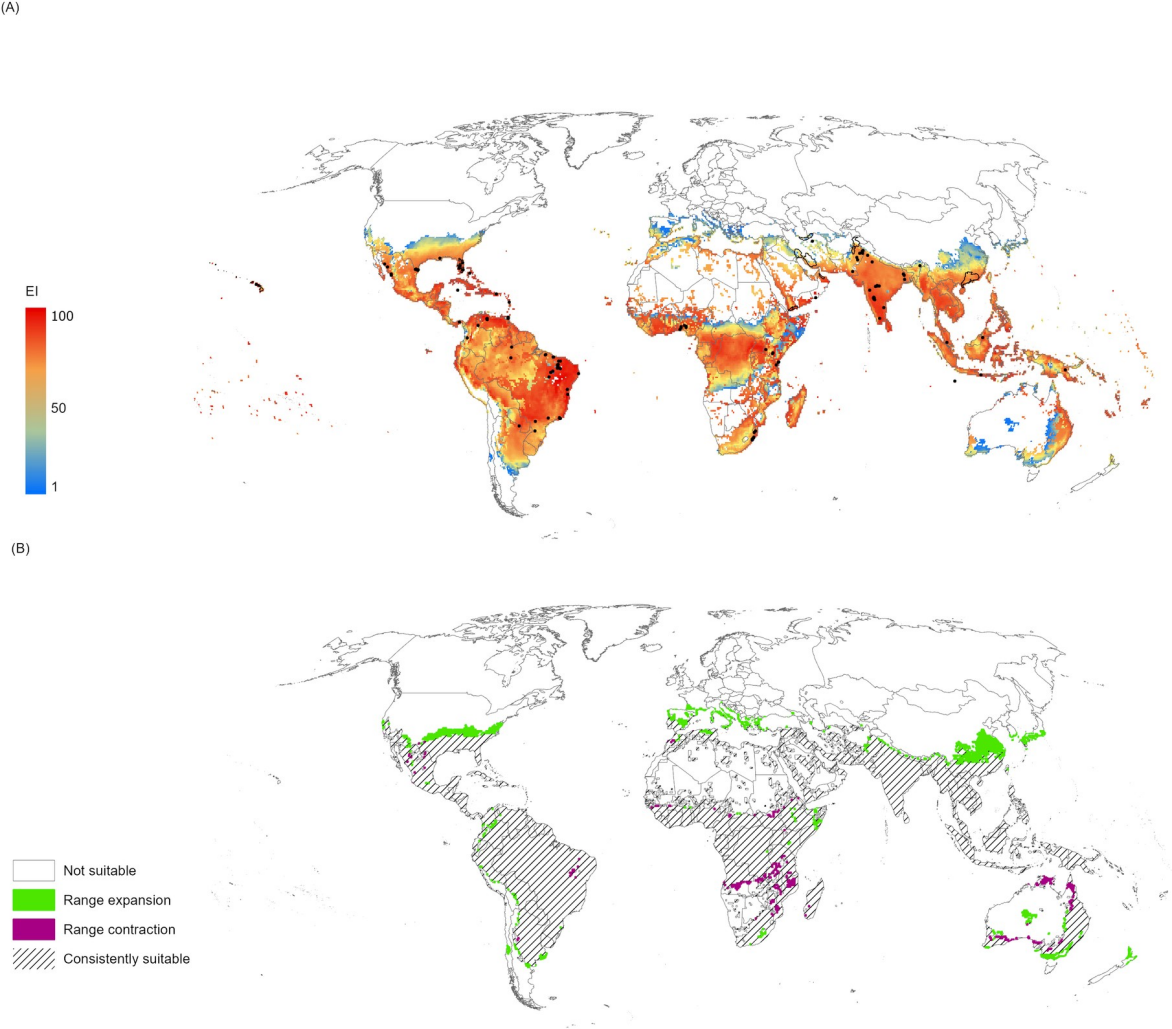
Α) Modelled global climate suitability for *Aleurocanthus woglumi* after the RCP8.5 ACCESS 1–0 model was applied for years 2040–2059, as a composite of natural rainfall and irrigation scenarios based on irrigation areas identified by Siebert et al. [64]. B) Forecast qualitative variation of ecoclimatic suitability between historical climate (Fig 1) and 2050 projected climate (Fig 2A) highlighting the expansion (current EI = 0, future EI>0) and contraction (current EI>0, future EI = 0) of suitable areas for the 2050 projection. The Ecoclimatic Index (EI) describes the overall climate suitability for population persistence, where 0 is unsuitable and 100 is year-round optimal conditions. This map was produced by the authors using ArcGIS Pro 2.7.1 software (@esri.com; no copyrighted material was used). Global irrigation areas [64] are used herein under a CC BY 4.0 license, with permission from Stefan Siebert, original copyright 2013. Boundary data for the countries of the world come from Natural Earth (@naturalearthdata.com; public domain) [65].

Suitable areas for pest establishment were quantified in terms of their expected future expansion or contraction under the RCP8.5 ACCESS1-0 model as compared to the areas modelled as suitable in the presence of irrigation under current climate projections (Fig 2B). The projected increase in suitable areas in comparison to those under current climate is relatively modest, with most of this expansion occurring within Europe. In Asia, North America, Oceania, and South America there are increases in the regions that are modelled with EI>0 for *A*. *woglumi* of 11, 15, 2, and 1%, respectively, while in Europe the potential distribution increases by 90%, though from a very low base of ≈300 000 km^2^ (Table 2). Future climate projection in Africa suggests a decrease of 2%.

In Asia, the model projects an overall moderate expansion in the potential range of *A*. *woglumi* (Figs 2B, S3A and S3B). In the Northern Hemisphere, especially in China, the potential range expands northward, although the expansion in India is limited because the present range is bounded by the foothills of the Himalayas. The short-term climate forecasts are insufficient to shift the range appreciably into this mountainous area. In South Korea and Japan, there is potential for future expansion in southern coastal areas.

According to the future climate scenario, winter minimum temperatures generally are expected to rise disproportionately faster in the northern latitudes [68–70]. In North America, the potential range of areas with EI>0 expands northward as depicted in Figs 2B, S4A and S4B. As a result, many southern states of the USA will likely become favorable for *A*. *woglumi*, although in specific regions within these states, the insect will probably experience heat stress but continue to be present at low levels.

In South America, a slight increase in climate suitability is projected for *A*. *woglumi* in the near future (Fig 2B). Major decreases in areas modelled as suitable are observed in the eastern part of Brazil but decreases are also visible in portions of the Amazon basin as well as in states of central and northern Brazil (Figs 2A, S5A and S5B). According to climate projections, by mid-century increases in temperature and associated decreases in soil water are projected to lead to gradual replacement of tropical forest by savanna in eastern Amazonia, as changes in precipitation patterns and the disappearance of glaciers are projected to significantly affect water availability [51,71]. These changes would likely be accompanied by a rise in dry and heat stress levels and the productivity of some important crops is projected to decrease [50]. As it is projected for other tropical regions, water stress, increased temperature and the impact of an increased incidence and abundance of old and new invasive pests, diseases and weeds will lead to agricultural decline [23].

In Africa, a slight decrease in the climatically suitable area is projected, but the range of ecoclimatic values remains relatively high. Some areas in northern Tunisia and Morocco with temperate climate are projected to remain climatically suitable for *A*. *woglumi* (Figs 2A, S6A and S6B). In areas in central and southern Africa such as Zambia, Zimbabwe, and neighboring countries there is a range contraction, and the areas where the range expands are restricted to the northern and eastern parts of Africa (Fig 2B).

In Europe, the potential distribution of *A*. *woglumi* expands northward under the future climate scenarios (Fig 2B). Due to temperature increases, the cold limitations in some areas could be overcome under future climate scenarios. At the same time, drought-affected regions are projected to increase in extent, especially in the Mediterranean Basin [50], and in combination with the temperature increases are projected to limit wide dispersal of the pest (Figs 2A, S7A and S7B).

In Oceania, the future climate simulations project that areas with EI>0 for *A*. *woglumi* will expand slightly (Fig 2B). In Australia, the model projects a progressive contraction of suitable regions, especially in the south and north-west. In New Zealand, Northland and parts of East Cape and the Bay of Plenty become favourable under the future climate projections, potentially exposing citrus and other horticultural industries to *A*. *woglumi* invasion risks (Figs 2A, S8A and S8B).

The use of a spatially explicit irrigation scenario provides a highly effective means of estimating the potential distribution of agricultural hosts and pests. It avoids the problem of adopting biologically implausible soil moisture parameters to “explain” the presence of a species in xeric environments, with the subsequent problem of then modeling all locations with a similar xeric climate as being suitable, severely diminishing model specificity.

Environmental suitability simulations for invasive species under current and future scenarios provide a strategic tool for risk managers to gain foresight into emerging pest invasion patterns, enabling them to develop effective control and surveillance strategies. Hence, the model presented in this study for *A*. *woglumi* can be used to estimate current and emerging risk areas to develop better procedures for early detection and improve resource allocation for adaptive management under climate change. The irreducible uncertainties in forecasting the emission patterns of greenhouse gases translate into substantial uncertainties in the extent and rate of change in climates. Model sensitivity analyses such as those we have performed should guide the development of contingency planning on the basis that the models may be right, but equally, they may under- or over-estimate the climatic changes. A prudent contingency plan will include a solid investment in surveillance, identifying and monitoring lead indicator variables that can signal if and when the plan needs to be activated, or at least be reconsidered in the light of shifting circumstances.

It is easy to identify low-cost, no regrets management options for *A*. *woglumi*. For example, in the case of *A*. *woglumi* biosecurity, while the present threat to New Zealand appears low due to excessive cold stress during winter, this protection appears vulnerable to climate change within the next thirty years. Unless costs are prohibitive, phytosanitary measures to prevent the spread of *A*. *woglumi* to New Zealand would seem reasonable under present climatic conditions, potentially avoiding seasonal market access issues if there were an incursion. These same measures would also help prevent the potential establishment of this pest in the future as the winter protection is eroded by a warming climate. Currently, with good reason, Australia maintains phytosanitary measures to prevent the entry and establishment of *A*. *woglumi*.

The present invasion threat posed by *A*. *woglumi* to Europe encompasses most of the citrus-growing regions in the Mediterranean Basin. The threat appears set to expand northwards with further climate change. It would seem prudent to try to prevent the establishment of *A*. *woglumi* in Europe, North Africa and the Middle East. The Nile Valley (Fig 1) poses an obvious invasion pathway towards Europe, alongside the movement of trade goods via shipping from sub-Saharan Africa via coastal Atlantic and Suez Canal routes. The expansion of irrigation in the Arabian Peninsula has created an additional set of stepping-stones for biological invasions to the north.

In North America, it would seem sensible to try to limit the spread of *A*. *woglumi* from Texas and Florida into Arizona and California where there are valuable citrus production areas. Under current climatic conditions, California and Arizona appear unsuitable for *A*. *woglumi* to persist, though with a warming climate this situation appears set to change. In contrast to Australia and New Zealand, where there are maritime borders, quarantine measures to restrict the movement of *A*. *woglumi* across state borders may be infeasible, cost-prohibitive, or politically unpalatable.

Given the difficulty and expense of eradication efforts, there may be value in undertaking pre-emptive biological control preparations, undertaking host specificity testing of candidate agents such as *Encarsia perplexa* and *Amitus hesperidum* [72,73]. Such Hymenopteran parasites have been proved economical and effective because they kept *A*. *woglumi* below damaging levels in much of its distribution range in several parts of the world like southern Florida [74], Trinidad & Tobago [72], Texas [75], Mexico [76] and Dominica [73].

In an exciting development, New Zealand has established conditional approval for the import and deployment of *Trissolcus japonicus*, a biological control agent for *Halyomorpha halys* [77]. The New Zealand Government recognised the invasion threat posed by *H*. *halys* and supported the testing and approval ahead of the arrival of *H*. *halys*. The foresight provided by the analyses in this paper could be used to justify such a programme for *A*. *woglumi* and provide a basis for countries to join together to complete host-specificity testing, taking advantage of economies of scale.

## Supporting information

S1 Fig Modelled global climate suitability for *Aleurocanthus woglumi* as a composite of natural rainfall and irrigation scenarios based on irrigation areas identified by Siebert et al. [64]. The Ecoclimatic Index (EI) describes the overall climate suitability for population persistence, where 0 is unsuitable and 100 is year-round optimal conditions. Black dots indicate location records for *A*. *woglumi* and black polygons the administrative areas where *A*. *woglumi* has been reported (S1 Table). Purple shaded areas are those that are modelled as suitable for *A*. *woglumi* only under the irrigation scenario. This map was produced by the authors using ArcGIS Pro 2.7.1 software (@esri.com; no copyrighted material was used). Global irrigation areas [64] are used herein under a CC BY 4.0 license, with permission from Stefan Siebert, original copyright 2013. Boundary data for the countries of the world come from Natural Earth (@naturalearthdata.com; public domain) [65], and provincial/regional boundary data from geoBoundaries (@geoboundaries.org) [66]. GeoBoundaries are used herein under a CC BY 4.0 license, with permission from Daniel Runfola, original copyright 2020.
(TIF)

S2 Fig CLIMEX global uncertainty analysis for the *Aleurocanthus woglumi* model under the natural rainfall scenario. The proportional model agreement (%) for sampled parameter uncertainty. This map was produced by the authors using ArcGIS Pro 2.7.1 software (@esri.com; no copyrighted material was used). Boundary data for the countries of the world come from Natural Earth (@naturalearthdata.com; public domain) [65].
(TIF)

S3 Fig Trends in area suitability A) under a historical climate scenario, represented by a composite of natural rainfall and irrigation scenarios based on irrigation areas identified by Siebert et al. [64], and B) after the RCP8.5 ACCESS 1–0 model scenario was applied for years 2040–2059, as a composite of natural rainfall and irrigation scenarios in Asia. The Ecoclimatic Index (EI) describes the overall climate suitability for population persistence, where 0 is unsuitable and 100 is year-round optimal conditions. This map was produced by the authors using ArcGIS Pro 2.7.1 software (@esri.com; no copyrighted material was used). Global irrigation areas [64] are used herein under a CC BY 4.0 license, with permission from Stefan Siebert, original copyright 2013. Boundary data for the countries of the world come from Natural Earth (@naturalearthdata.com; public domain) [65].
(TIF)

S4 Fig Trends in climate suitability A) under a historical climate scenario, represented by a composite of natural rainfall and irrigation scenarios based on irrigation areas identified by Siebert et al. [64], and B) after the RCP8.5 ACCESS 1–0 model scenario was applied for years 2040–2059, as a composite of natural rainfall and irrigation scenarios in North America. The Ecoclimatic Index (EI) describes the overall climate suitability for population persistence, where 0 is unsuitable and 100 is year-round optimal conditions. This map was produced by the authors using ArcGIS Pro 2.7.1 software (@esri.com; no copyrighted material was used). Global irrigation areas [64] are used herein under a CC BY 4.0 license, with permission from Stefan Siebert, original copyright 2013. Boundary data for the countries of the world come from Natural Earth (@naturalearthdata.com; public domain) [65].
(TIF)

S5 Fig Trends in climate suitability A) under a historical climate scenario, represented by a composite of natural rainfall and irrigation scenarios based on irrigation areas identified by Siebert et al. [64], and B) after the RCP8.5 ACCESS 1–0 model scenario was applied for years 2040–2059, as a composite of natural rainfall and irrigation scenarios in South America. The Ecoclimatic Index (EI) describes the overall climate suitability for population persistence, where 0 is unsuitable and 100 is year-round optimal conditions. This map was produced by the authors using ArcGIS Pro 2.7.1 software (@esri.com; no copyrighted material was used). Global irrigation areas [64] are used herein under a CC BY 4.0 license, with permission from Stefan Siebert, original copyright 2013. Boundary data for the countries of the world come from Natural Earth (@naturalearthdata.com; public domain) [65].
(TIF)

S6 Fig Trends in climate suitability A) under a historical climate scenario, represented by a composite of natural rainfall and irrigation scenarios based on irrigation areas identified by Siebert et al. [64], and B) after the RCP8.5 ACCESS 1–0 model scenario was applied for years 2040–2059, as a composite of natural rainfall and irrigation scenarios in Africa. The Ecoclimatic Index (EI) describes the overall climate suitability for population persistence, where 0 is unsuitable and 100 is year-round optimal conditions. This map was produced by the authors using ArcGIS Pro 2.7.1 software (@esri.com; no copyrighted material was used). Global irrigation areas [64] are used herein under a CC BY 4.0 license, with permission from Stefan Siebert, original copyright 2013. Boundary data for the countries of the world come from Natural Earth (@naturalearthdata.com; public domain) [65].
(TIF)

S7 Fig Trends in climate suitability A) under a historical climate scenario, represented by a composite of natural rainfall and irrigation scenarios based on irrigation areas identified by Siebert et al. [64], and B) after the RCP8.5 ACCESS 1–0 model scenario was applied for years 2040–2059, as a composite of natural rainfall and irrigation scenarios in Europe. The Ecoclimatic Index (EI) describes the overall climate suitability for population persistence, where 0 is unsuitable and 100 is year-round optimal conditions. This map was produced by the authors using ArcGIS Pro 2.7.1 software (@esri.com; no copyrighted material was used). Global irrigation areas [64] are used herein under a CC BY 4.0 license, with permission from Stefan Siebert, original copyright 2013. Boundary data for the countries of the world come from Natural Earth (@naturalearthdata.com; public domain) [65].
(TIF)

S8 Fig Trends in climate suitability A) under a historical climate scenario, represented by a composite of natural rainfall and irrigation scenarios based on irrigation areas identified by Siebert et al. [64], and B) after the RCP8.5 ACCESS 1–0 model scenario was applied for years 2040–2059, as a composite of natural rainfall and irrigation scenarios in Oceania. The Ecoclimatic Index (EI) describes the overall climate suitability for population persistence, where 0 is unsuitable and 100 is year-round optimal conditions. This map was produced by the authors using ArcGIS Pro 2.7.1 software (@esri.com; no copyrighted material was used). Global irrigation areas [64] are used herein under a CC BY 4.0 license, with permission from Stefan Siebert, original copyright 2013. Boundary data for the countries of the world come from Natural Earth (@naturalearthdata.com; public domain) [65].
(TIF)

S1 Table Known distribution records of *Aleurocanthus woglumi*: A) known locations (points) and B) known administrative areas (polygons; coordinates are for the polygon centroids).
(PDF)

S2 Table CLIMEX parameter sensitivity values for *Aleurocanthus woglumi* parameters listed in Table 1, as applied to the CM30 1995H V2 global dataset under a natural rainfall scenario. (PDF)
