# Supplementary figures and images for: Potential global distribution of Aleurocanthus woglumi considering climate change and irrigation
Source: PLoS One. 2021 Dec 20;16(12):e0261626. doi: 10.1371/journal.pone.0261626 (PMC8687537; doi:10.1371/journal.pone.0261626)

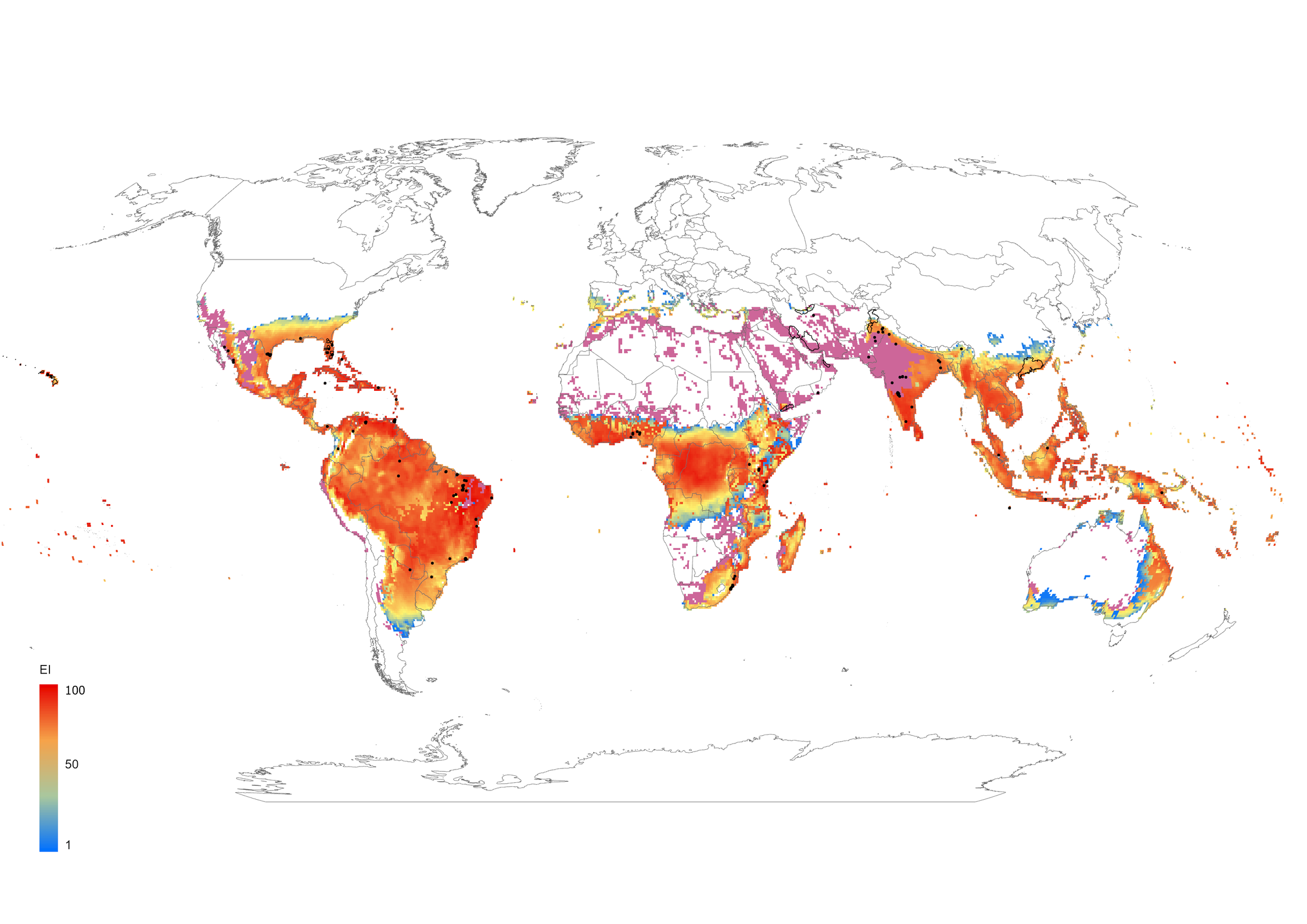

Supplement: S1 Fig — The Ecoclimatic Index (EI) describes the overall climate suitability for population persistence, where 0 is unsuitable and 100 is year-round optimal conditions. Black dots indicate location records for A. woglumi and black polygons the administrative areas where A. woglumi has been reported (S1 Table). Purple shaded areas are those that are modelled as suitable for A. woglumi only under the irrigation scenario. This map was produced by the authors using ArcGIS Pro 2.7.1 software (@esri.com; no copyrighted material was used). Global irrigation areas [64] are used herein under a CC BY 4.0 license, with permission from Stefan Siebert, original copyright 2013. Boundary data for the countries of the world come from Natural Earth (@naturalearthdata.com; public domain) [65], and provincial/regional boundary data from geoBoundaries (@geoboundaries.org) [66]. GeoBoundaries are used herein under a CC BY 4.0 license, with permission from Daniel Runfola, original copyright 2020. (TIF) [file pone.0261626.s001.tif]

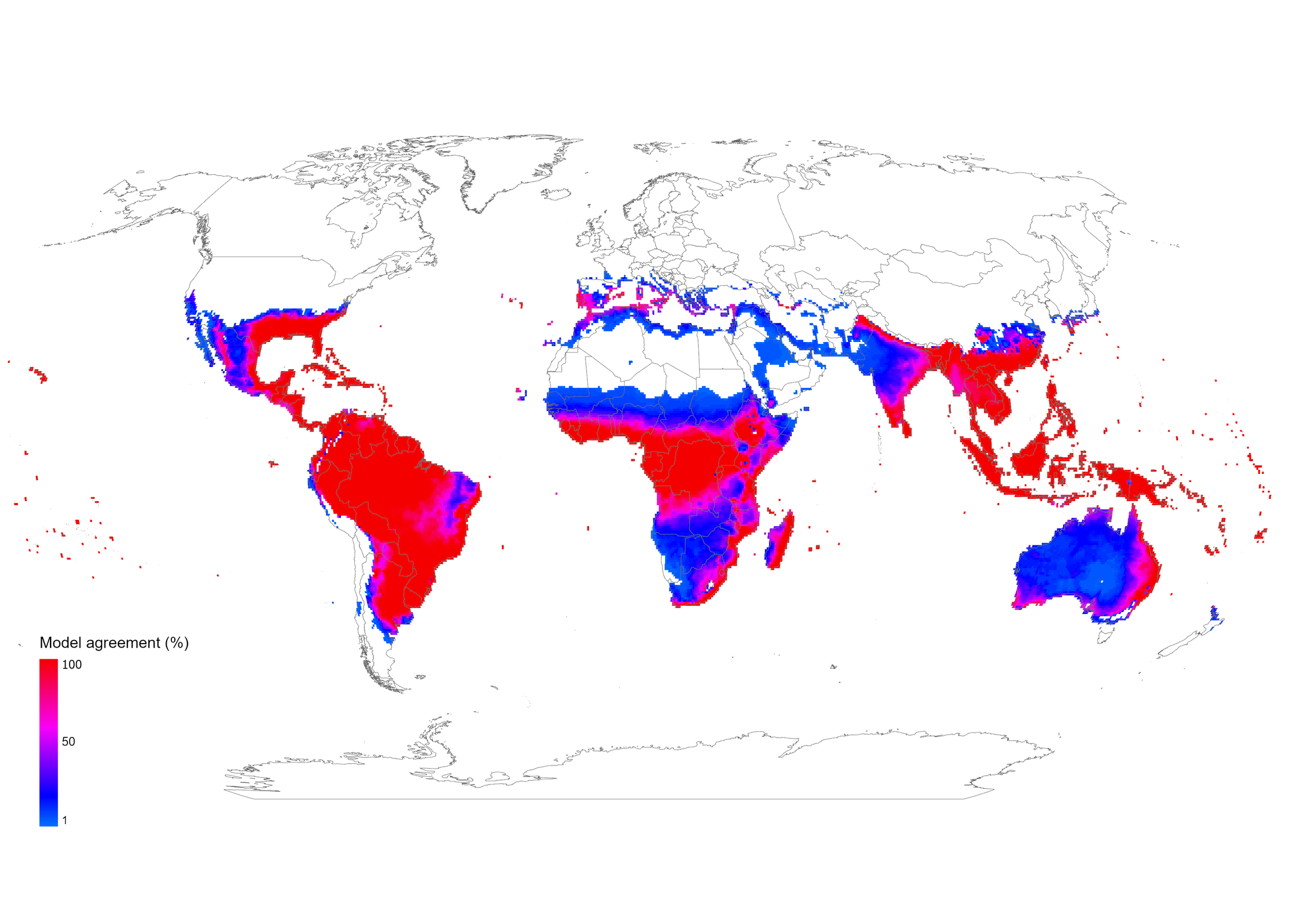

Supplement: S2 Fig — The proportional model agreement (%) for sampled parameter uncertainty. This map was produced by the authors using ArcGIS Pro 2.7.1 software (@esri.com; no copyrighted material was used). Boundary data for the countries of the world come from Natural Earth (@naturalearthdata.com; public domain) [65]. (TIF) [file pone.0261626.s002.tif]

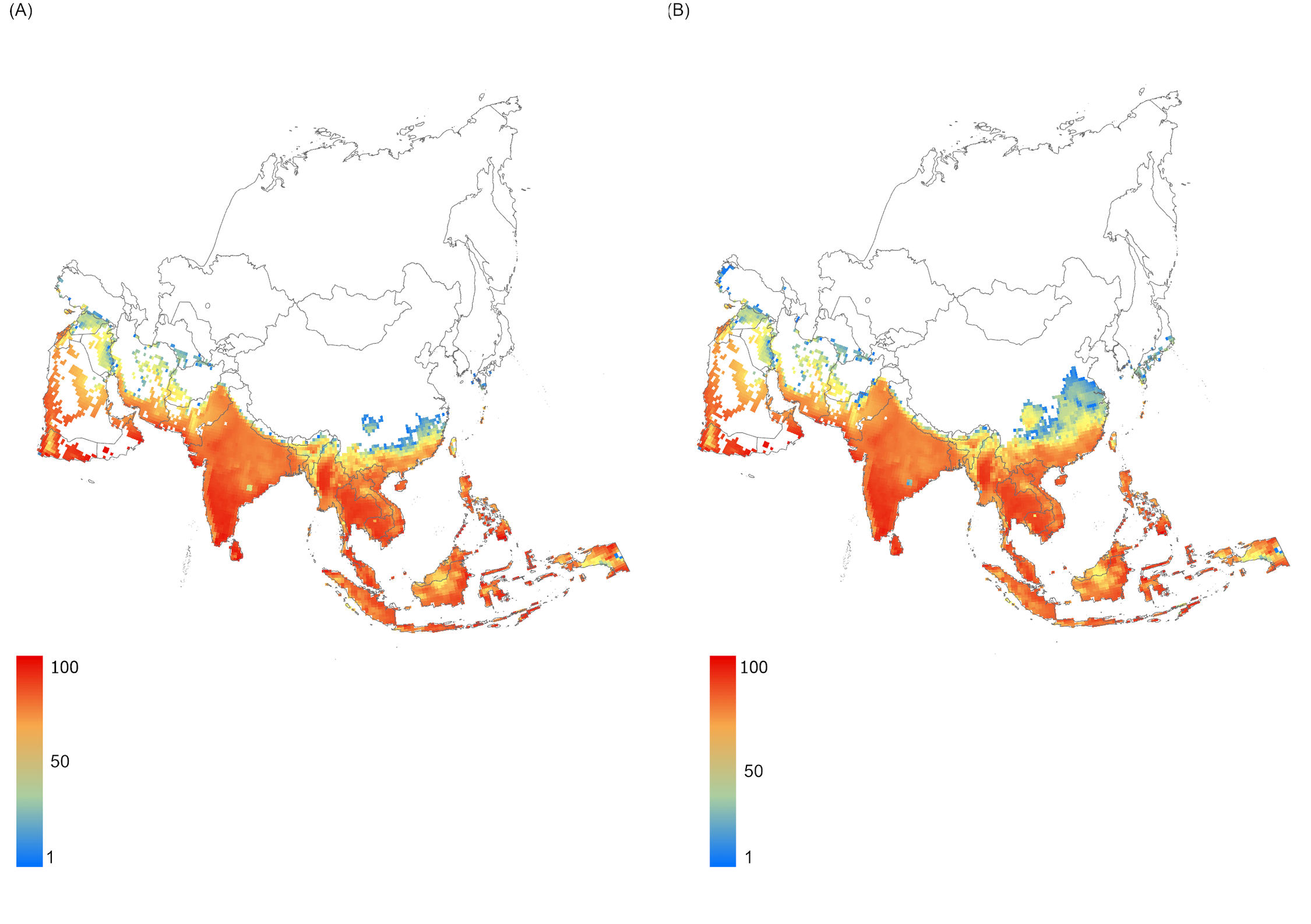

Supplement: S3 Fig — Trends in area suitability A) under a historical climate scenario, represented by a composite of natural rainfall and irrigation scenarios based on irrigation areas identified by Siebert et al. [64], and B) after the RCP8.5 ACCESS 1–0 model scenario was applied for years 2040–2059, as a composite of natural rainfall and irrigation scenarios in Asia. The Ecoclimatic Index (EI) describes the overall climate suitability for population persistence, where 0 is unsuitable and 100 is year-round optimal conditions. This map was produced by the authors using ArcGIS Pro 2.7.1 software (@esri.com; no copyrighted material was used). Global irrigation areas [64] are used herein under a CC BY 4.0 license, with permission from Stefan Siebert, original copyright 2013. Boundary data for the countries of the world come from Natural Earth (@naturalearthdata.com; public domain) [65]. (TIF) [file pone.0261626.s003.tif]

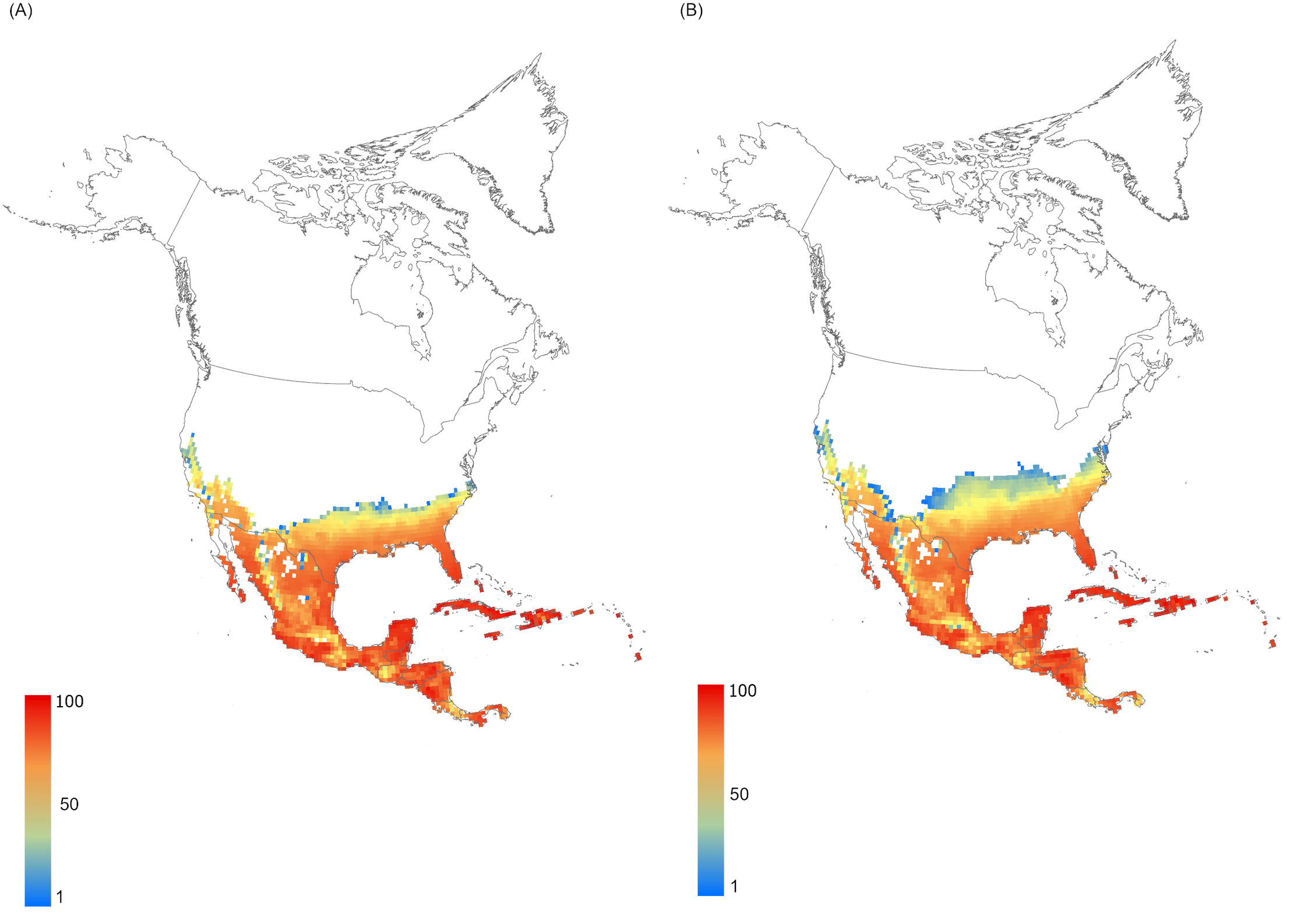

Supplement: S4 Fig — Trends in climate suitability A) under a historical climate scenario, represented by a composite of natural rainfall and irrigation scenarios based on irrigation areas identified by Siebert et al. [64], and B) after the RCP8.5 ACCESS 1–0 model scenario was applied for years 2040–2059, as a composite of natural rainfall and irrigation scenarios in North America. The Ecoclimatic Index (EI) describes the overall climate suitability for population persistence, where 0 is unsuitable and 100 is year-round optimal conditions. This map was produced by the authors using ArcGIS Pro 2.7.1 software (@esri.com; no copyrighted material was used). Global irrigation areas [64] are used herein under a CC BY 4.0 license, with permission from Stefan Siebert, original copyright 2013. Boundary data for the countries of the world come from Natural Earth (@naturalearthdata.com; public domain) [65]. (TIF) [file pone.0261626.s004.tif]

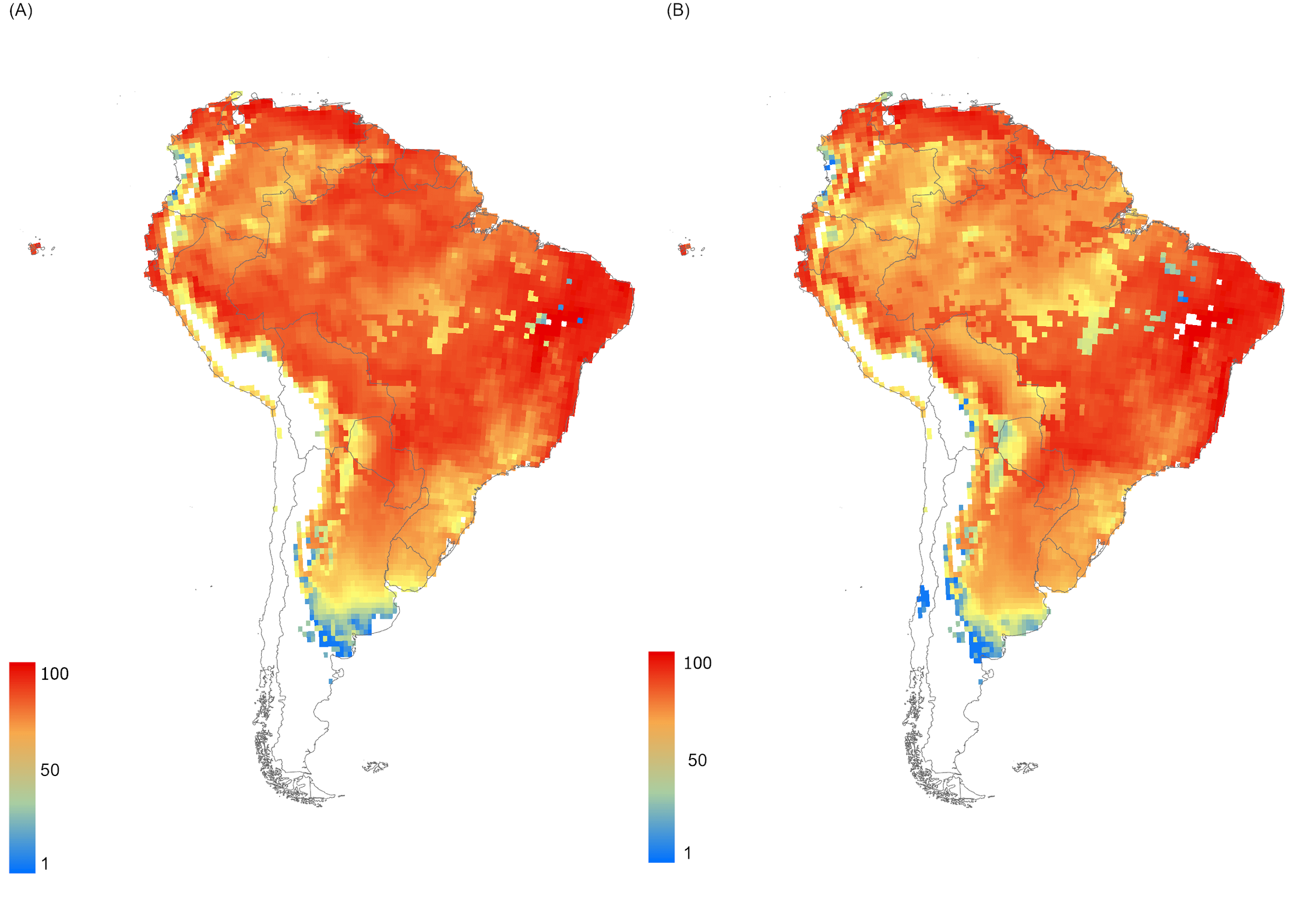

Supplement: S5 Fig — Trends in climate suitability A) under a historical climate scenario, represented by a composite of natural rainfall and irrigation scenarios based on irrigation areas identified by Siebert et al. [64], and B) after the RCP8.5 ACCESS 1–0 model scenario was applied for years 2040–2059, as a composite of natural rainfall and irrigation scenarios in South America. The Ecoclimatic Index (EI) describes the overall climate suitability for population persistence, where 0 is unsuitable and 100 is year-round optimal conditions. This map was produced by the authors using ArcGIS Pro 2.7.1 software (@esri.com; no copyrighted material was used). Global irrigation areas [64] are used herein under a CC BY 4.0 license, with permission from Stefan Siebert, original copyright 2013. Boundary data for the countries of the world come from Natural Earth (@naturalearthdata.com; public domain) [65]. (TIF) [file pone.0261626.s005.tif]

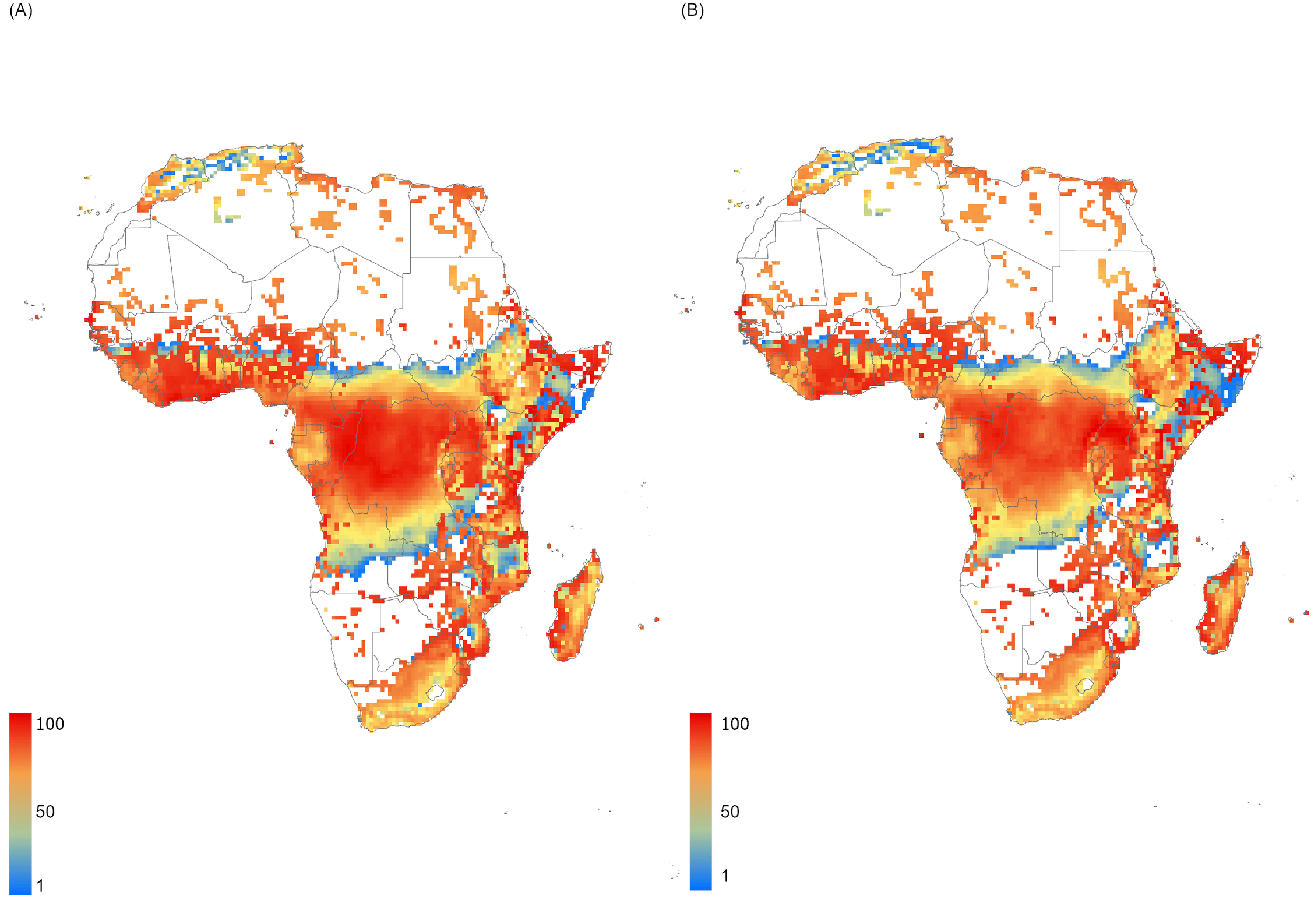

Supplement: S6 Fig — Trends in climate suitability A) under a historical climate scenario, represented by a composite of natural rainfall and irrigation scenarios based on irrigation areas identified by Siebert et al. [64], and B) after the RCP8.5 ACCESS 1–0 model scenario was applied for years 2040–2059, as a composite of natural rainfall and irrigation scenarios in Africa. The Ecoclimatic Index (EI) describes the overall climate suitability for population persistence, where 0 is unsuitable and 100 is year-round optimal conditions. This map was produced by the authors using ArcGIS Pro 2.7.1 software (@esri.com; no copyrighted material was used). Global irrigation areas [64] are used herein under a CC BY 4.0 license, with permission from Stefan Siebert, original copyright 2013. Boundary data for the countries of the world come from Natural Earth (@naturalearthdata.com; public domain) [65]. (TIF) [file pone.0261626.s006.tif]

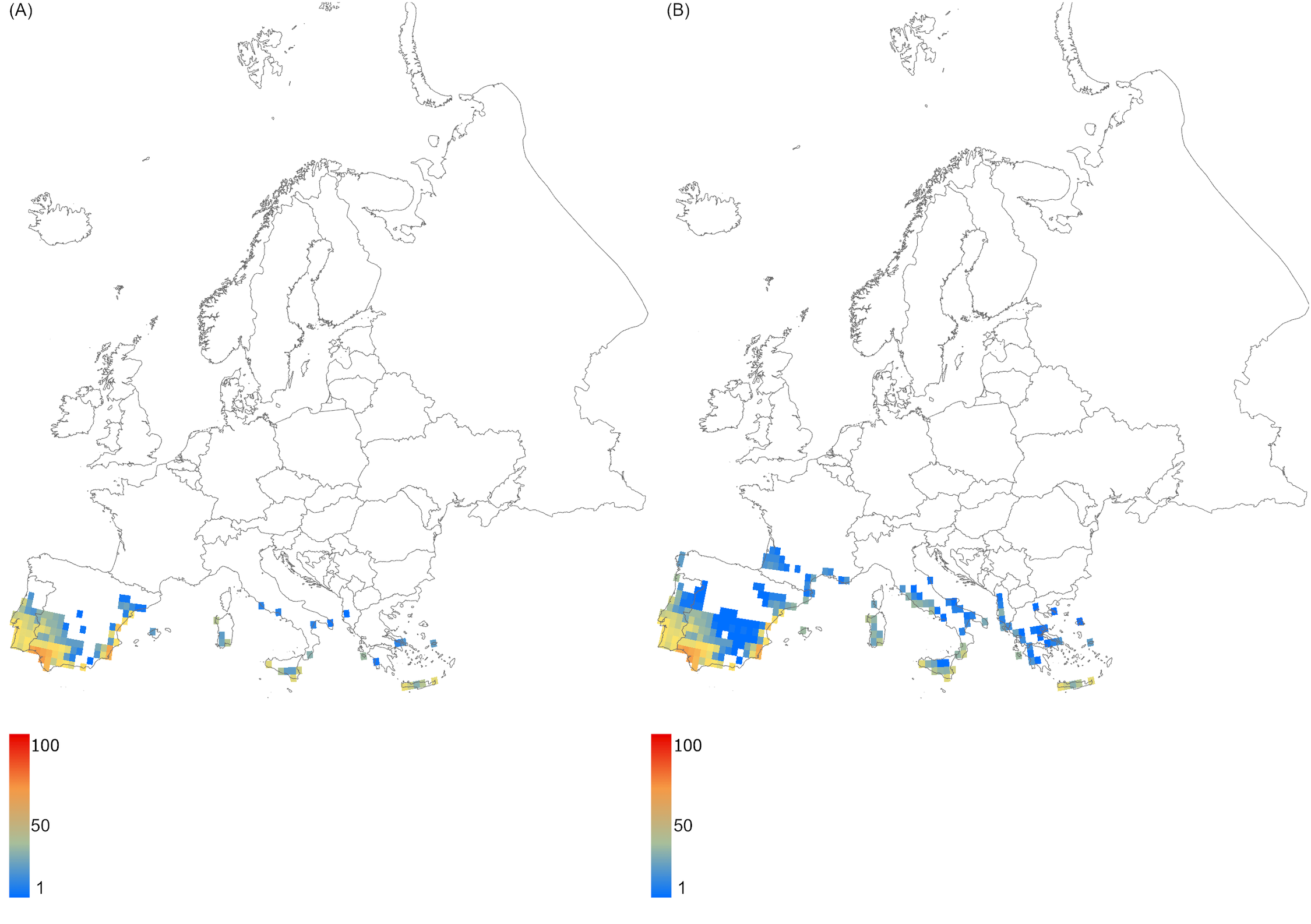

Supplement: S7 Fig — Trends in climate suitability A) under a historical climate scenario, represented by a composite of natural rainfall and irrigation scenarios based on irrigation areas identified by Siebert et al. [64], and B) after the RCP8.5 ACCESS 1–0 model scenario was applied for years 2040–2059, as a composite of natural rainfall and irrigation scenarios in Europe. The Ecoclimatic Index (EI) describes the overall climate suitability for population persistence, where 0 is unsuitable and 100 is year-round optimal conditions. This map was produced by the authors using ArcGIS Pro 2.7.1 software (@esri.com; no copyrighted material was used). Global irrigation areas [64] are used herein under a CC BY 4.0 license, with permission from Stefan Siebert, original copyright 2013. Boundary data for the countries of the world come from Natural Earth (@naturalearthdata.com; public domain) [65]. (TIF) [file pone.0261626.s007.tif]

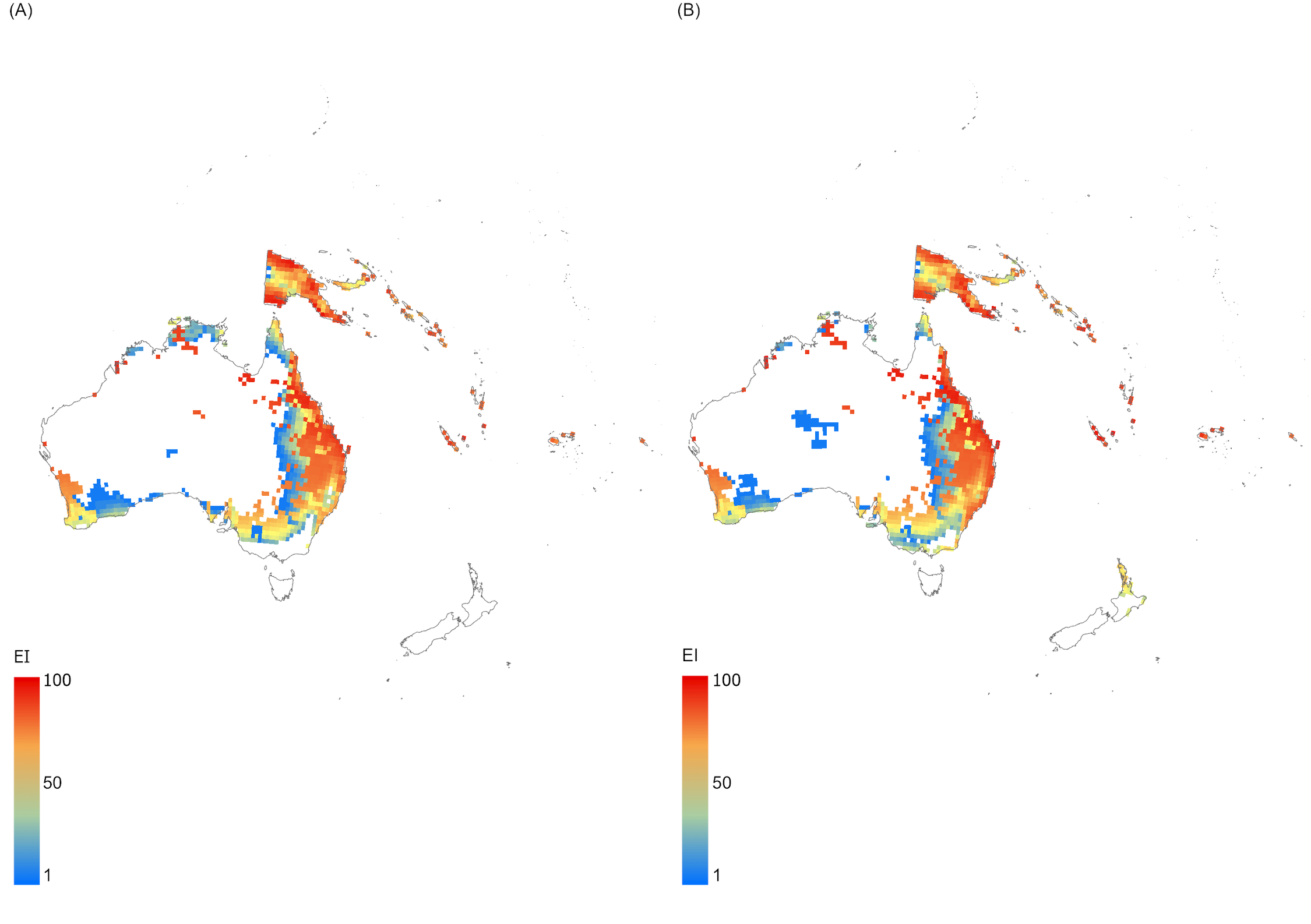

Supplement: S8 Fig — Trends in climate suitability A) under a historical climate scenario, represented by a composite of natural rainfall and irrigation scenarios based on irrigation areas identified by Siebert et al. [64], and B) after the RCP8.5 ACCESS 1–0 model scenario was applied for years 2040–2059, as a composite of natural rainfall and irrigation scenarios in Oceania. The Ecoclimatic Index (EI) describes the overall climate suitability for population persistence, where 0 is unsuitable and 100 is year-round optimal conditions. This map was produced by the authors using ArcGIS Pro 2.7.1 software (@esri.com; no copyrighted material was used). Global irrigation areas [64] are used herein under a CC BY 4.0 license, with permission from Stefan Siebert, original copyright 2013. Boundary data for the countries of the world come from Natural Earth (@naturalearthdata.com; public domain) [65]. (TIF) [file pone.0261626.s008.tif]
