# Supplementary material for: Potential global distribution of Aleurocanthus woglumi considering climate change and irrigation
Source: PLoS One. 2021 Dec 20;16(12):e0261626. doi: 10.1371/journal.pone.0261626 (PMC8687537; doi:10.1371/journal.pone.0261626)
Supplement: S1 Table — Known distribution records of Aleurocanthus woglumi: A) known locations (points) and B) known administrative areas (polygons; coordinates are for the polygon centroids). (PDF) [file pone.0261626.s009.pdf]

S1 Table. Known distribution records of *Aleurocanthus woglumi*.

A) Known locations (points).

Continent	Location	Latitude (DD)	Longitude (DD)	Reference number
North America	USA, Florida, Ft. Lauderdale	26.131380	-80.130346	1
North America	USA, Florida, Lee, Lehigh Acres	26.624367	-81.624256	2
North America	USA, Florida, Lee, Fort Myers	26.640208	-81.874172	2
North America	USA, Florida, Highlands, Sun 'n Lake	27.531286	-81.527542	2
North America	USA, Florida, Brevard, Merritt Island-Cocoa	28.379731	-80.744281	2
North America	USA, Florida, Broward, Davie	26.062870	-80.233100	3
North America	USA, Florida, Broward, Davie	26.085030	-80.239890	3
North America	USA, Texas, Hidalgo, Mission	26.231723	-98.309400	4,5
North America	USA, Texas, Cameron, Bayview	26.129352	-97.399292	5
North America	USA, Texas, Hidalgo, Donna	26.169453	-98.051858	5
North America	USA, Texas, Mercedes	26.143981	-97.888757	5
North America	USA, Texas, Hidalgo, Edinburg	26.314059	-98.137729	5
North America	USA, Texas, Hidalgo, La Joya	26.241150	-98.484842	6
North America	USA, Texas, Cameron, Harlingen	26.162017	-97.757676	6
North America	USA, Texas, Cameron, Brownsville	25.899346	-97.513485	6
North America	USA, Texas, Hidalgo, Weslaco	26.159170	-97.990560	3
North America	USA, Hawaii, Honolulu	21.306940	-157.858330	3
North America	USA, Louisiana, New Orleans	29.954600	-90.075100	3
North America	Mexico, Sinaloa, Culiacán, El Dorado	24.302589	-107.333211	7
North America	Mexico, Sinaloa, Culiacán	24.804160	-107.431110	3
North America	Mexico, Sonora, Nacozari de García, Navojoa	27.063960	-109.440050	3
North America	Mexico, Sonora, Empalme	27.965210	-110.810380	3
North America	Mexico, Sonora, Navojoa	27.071670	-109.446390	3
South America	Brazil, Rio de Janeiro, Cachoeiras de Macacu, Papucaia	-22.591731	-42.745311	8
South America	Brazil, Rio de Janeiro, Cachoeiras de Macacu, Papucaia	-22.585686	-42.722311	8
South America	Brazil, Rio de Janeiro, Macacu	-22.592669	-42.744278	9

South America	Brazil, Rio de Janeiro, Macacu	-22.512319	-42.822921	9
South America	Brazil, Rio de Janeiro, Macacu	-22.511618	-42.821124	9
South America	Brazil, Rio de Janeiro, Macacu	-22.590469	-42.740026	9
South America	Brazil, Rio de Janeiro, Macacu	-22.620695	-42.829196	9
South America	Brazil, Rio de Janeiro, Guapimirim	-22.542840	-42.929009	9
South America	Brazil, Rio de Janeiro, Itaboraí	-22.796206	-42.834856	9
South America	Brazil, Rio de Janeiro, Itaboraí	-22.658009	-42.771928	9
South America	Brazil, Rio de Janeiro, Rio Bonito	-22.771758	-42.670924	9
South America	Brazil, Rio de Janeiro, Rio Bonito	-22.777675	-42.672401	9
South America	Brazil, Rio de Janeiro, Rio Bonito	-22.771683	-42.668238	9
South America	Brazil, Rio de Janeiro, Rio Bonito	-22.787585	-42.661103	9
South America	Brazil, Rio de Janeiro, Rio Bonito	-22.772197	-42.671296	9
South America	Brazil, Rio de Janeiro, Tanguá	-22.754344	-42.721090	9
South America	Brazil, Rio de Janeiro, Tanguá	-22.753769	-42.708104	9
South America	Brazil, Rio de Janeiro, Tanguá	-22.772022	-42.676069	9
South America	Brazil, Rio de Janeiro, Tanguá	-22.753185	-42.707646	9
South America	Brazil, São Paulo, Artur Nogueira	-22.604383	-47.152850	10
South America	Brazil, São Paulo, Artur Nogueira, Sao Bento	-22.573056	-47.172500	11
South America	Brazil, Pará, Capitão Poço	-1.810556	-47.193889	12
South America	Brazil, Pará, Capitão Poço	-1.754167	-47.125000	13
South America	Brazil, Paraná, Mandaguaçu	-23.648303	-52.140714	14
South America	Brazil, Santa Catarina, Coronel Freitas	-26.914833	-52.721250	15
South America	Brazil, Maranhão, Paço do Lumiar	-2.501361	-44.073000	16
South America	Brazil, Maranhão, Limão Rugoso	-2.584444	-44.209250	17
South America	Brazil, Maranhão, São José de Ribamar	-2.551333	-44.218778	18
South America	Brazil, Roraima, Caroebe	0.763917	-59.709306	19
South America	Brazil, Amazonas, Manaus	-2.853500	-60.044611	20
South America	Brazil, Bahia, Laje	-13.163694	-39.335722	21
South America	Brazil, Bahia, Laje	-13.202417	-39.334528	21
South America	Brazil, Bahia, Ilhéus	-14.797528	-39.172500	21
South America	Brazil, Piauí, Recife	-8.058044	-34.881567	22

South America	Brazil, Piauí, Altos	-5.041597	-42.459760	23
South America	Brazil, Piauí, Amarante	-6.244160	-42.841728	23
South America	Brazil, Piauí, Angical do Piauí	-6.087157	-42.733478	23
South America	Brazil, Piauí, Barro Duro	-5.825648	-42.513842	23
South America	Brazil, Piauí, Esperantina	-3.891326	-42.228613	23
South America	Brazil, Piauí, Lagoa do Piauí	-5.420440	-42.644407	23
South America	Brazil, Piauí, Piracuruca	-3.945557	-41.702870	23
South America	Brazil, Piauí, Regeneração	-6.241102	-42.689514	23
South America	Brazil, Piauí, Ribeiro Gonçalves	-7.563541	-45.244217	23
South America	Brazil, Piauí, Santa Filomena	-9.113949	-45.913817	23
South America	Brazil, Piauí, Teresina	-5.096081	-42.773167	23
South America	Brazil, Piauí, Uruçuí	-7.237483	-44.559667	23
South America	Brazil, Piauí, Valença do Piauí	-6.420728	-41.767213	23
South America	Colombia, Valle del Cauca, Cerrito	3.683330	-76.316660	3
South America	Bahamas, New Providence Island, Nassau	25.048519	-77.365922	24
South America	Cayman Islands, Grand Cayman Island, North Side	19.351003	-81.210706	25
South America	Dominica, Saint David, Castle Bruce	15.442318	-61.257197	26
South America	Panama, Ancon, Canal Zone	8.957280	-79.549440	3
South America	Puerto Rico	18.240220	-66.426100	3
South America	Trinidad & Tobago, Victoria County, Moruga	10.088794	-61.281311	27
South America	Trinidad & Tobago, Couva-Tabaquite-Talparo, Freeport	10.446256	-61.402225	27
South America	Trinidad & Tobago, Penal-Debe, Penal	10.164847	-61.438150	27
South America	Trinidad & Tobago, Sangre Grande, Cumuto	10.584481	-61.207203	27
South America	Trinidad & Tobago, Todds Road	10.482314	-61.323631	27
South America	Trinidad & Tobago, Tableland	10.272969	-61.262431	27
South America	Venezuela, Yaracuy, Yaritagua	10.067463	-69.114237	28
South America	Venezuela, Lara, Cabudare	10.005812	-69.235675	28
South America	Venezuela, Sarare	9.791028	-69.151459	28
South America	Venezuela, Táchira, San Antonio del Táchira	7.815717	-72.450659	28
South America	Argentina, Formosa Pilagá, Tres Lagunas	-25.267406	-58.502195	29
Asia	Bangladesh, Rajshahi	24.372746	88.606698	30

Asia	Bangladesh, Chapainawabganj	24.741214	88.289157	30
Asia	India, northern Karnataka, Vijayapur, Aliyabad	16.891367	75.795059	31
Asia	India, northern Karnataka, Vijayapur, Nagtan	16.930217	75.848705	31
Asia	India, northern Karnataka, Vijayapur, Aheri	16.875270	75.878660	31
Asia	India, northern Karnataka, Vijayapur, Atharga	16.985039	75.886217	31
Asia	India, northern Karnataka, Vijayapur, Tamba	16.995717	75.995214	31
Asia	India, northern Karnataka, Vijayapur, Tadavalaga	17.078953	75.929967	31
Asia	India, northern Karnataka, Vijayapur, Devara Hipparagi	16.816314	76.069122	31
Asia	India, northern Karnataka, Vijayapur, Kannolli	16.857041	76.153655	31
Asia	India, northern Karnataka, Vijayapur, Rudagi	16.432814	76.072583	31
Asia	India, northern Karnataka, Vijayapur, Nalatawada	16.248894	76.289236	31
Asia	India, northern Karnataka, Vijayapur, Dhavalagi	16.433007	76.119997	31
Asia	India, northern Karnataka, Vijayapur, Basavana Bagewadi	16.566177	75.972585	31
Asia	India, northern Karnataka, Vijayapur, Huvina Hipparagi	16.552814	76.074039	31
Asia	India, northern Karnataka, Vijayapur, Hunashyal	16.557681	76.033039	31
Asia	India, Tamil Nadu, Theni, Kumbakarai	10.135825	77.557489	32
Asia	India, Maharashtra, Darjeeling, Regional Research Sub-Station, Pedong of Bidhan Chandra Krishi Vishwavidyalaya	22.945146	88.533746	33
Asia	India, Maharashtra, Amravati	20.931228	77.734286	34
Asia	India, Maharashtra, Wardha	20.745833	78.603661	34
Asia	India, Maharashtra, Amravati, Morshi, Pala	20.840525	77.706994	35
Asia	India, Maharashtra, Akola	20.702244	77.025654	36
Asia	India, Maharashtra, Rahuri	19.396252	74.662204	36
Asia	India, Punjab, Ludhiana	30.930328	75.910119	37
Asia	India, Assam, Tinsukia	27.520741	95.355044	37
Asia	India, Tamil Nadu, Periyakulam	10.116354	77.541734	37
Asia	India, Andhra Pradesh, Tirupati	13.620049	79.374788	37
Asia	India, New Delhi, North Campus, New Delhi University	28.689330	77.214112	38
Asia	Indonesia, Bali, Gianyar Regency	-8.425369	115.259730	39
Asia	Iran, Semnan, Shahrood	35.500000	55.500000	40
Asia	Malaysia, Sarawak, Bario	3.808026	115.517273	41

Asia	Malaysia, Melaka	2.196000	102.24050	3
Asia	Oman, Dhofar, Al-Haffa	17.008959	54.108260	42
Asia	Oman, Dhofar, Salalah	17.015819	54.070913	42
Asia	Oman, Dhofar, Al-Wadi	17.008106	54.061495	42
Asia	Pakistan, Punjab, Gujranwala	32.213550	74.186397	7
Asia	Pakistan, Sindh, Mirpur Khas	25.507484	69.004443	7
Asia	Pakistan, Punjab, Lahore	31.525773	74.242759	7
Asia	Pakistan, Faisalabad	31.500000	73.013889	43
Asia	Pakistan, Sialkot	32.500000	74.516667	43
Asia	Pakistan, Multan	30.250000	71.600000	43
Asia	Pakistan, Bahawalpur	29.400000	71.666667	43
Asia	Christmas Island, Rainforest site "Pink House"	-10.492756	105.647405	44
Asia	Papua New Guinea, Morobe, Bundun	-6.850619	146.617222	45
Africa	Eswatini (former Swaziland), Lubombo, Big Bend	-26.792364	31.935056	46
Africa	Kenya, Machakos, Khayewa	-1.485908	37.351806	47
Africa	Kenya, Machakos, Kithimani	-1.168919	37.451767	47
Africa	Kenya, Machakos, Khayewa	-1.469483	37.356017	47
Africa	Kenya, Machakos, Khayewa	-1.423167	37.336167	47
Africa	Kenya, Machakos, Kithimani	-1.192483	37.449417	47
Africa	Kenya, Machakos, Kithimani	-1.182733	37.430900	47
Africa	Kenya, Machakos, Kithimani	-1.156350	37.435317	47
Africa	Kenya, Kwale, Matuga	-4.159000	39.597910	48
Africa	Kenya, Kisumu, Kibos	-0.065071	34.812650	48
Africa	Kenya, Kwale, Ukunda	-4.288362	39.567378	48
Africa	Nigeria, Lagos, Badagry	6.432622	2.874788	49
Africa	Nigeria, Ogun, Abeokuta	7.154147	3.386648	49
Africa	Nigeria, Oyo, Igbo-Ora	7.431333	3.308815	49
Africa	Nigeria, Osun, Osogbo	7.785678	4.542604	49
Africa	Nigeria, Ekiti, Ikere-Ekiti	7.487004	5.241104	49
Africa	Nigeria, Ondo, Idanre	7.091634	5.140163	49
Africa	South Africa, KwaZulu-Natal, Durban	-29.855475	31.025078	46

Africa	South Africa, KwaZulu-Natal, Stanger (now KwaDukuza)	-29.337803	31.283347	46
Africa	South Africa, KwaZulu-Natal, Gingindlovu	-29.026836	31.582975	46
Africa	South Africa, KwaZulu-Natal, Eshowe	-28.884769	31.462783	46
Africa	South Africa, KwaZulu-Natal, Nkwalini	-28.849254	31.499350	46
Africa	South Africa, KwaZulu-Natal, Pongola	-27.385097	31.619044	46
Africa	South Africa, KwaZulu-Natal, Malvern	-29.886153	30.919742	50
Africa	South Africa, KwaZulu-Natal, Verulam	-29.652128	31.039244	50
Africa	South Africa, KwaZulu-Natal, Oakford	-29.596116	31.014999	50
Africa	Tanzania, Tanga, Muheza	-5.165161	38.809807	51

References

1. Nguyen R, Hamon AB, Fasulo TR. Citrus blackfly, *Aleurocanthus woglumi*. Gainesville Florida Department of Agriculture & Consumer Service - Division of Plant Industry. 1998;3. EENY-42. Available from: https://edis.ifas.ufl.edu/pdf%5CIN%5CIN19900.pdf
2. Nguyen R, Brazzel JR, Poucher C. Population density of the citrus blackfly, *Aleurocanthus woglumi* Ashby (Homoptera: Aleyrodidae), and its parasites in urban Florida in 1979-1981. Environ Entomol. 1983;12: 878–884. doi: 10.1093/ee/12.3.878
3. Available from: http://research.amnh.org/pbi/heteropteraspeciespage/speciesdetails.php?fromall=fromall&speciesid=77922&genusid=77913 [accessed 16 October 2020].
4. Everitt JH, Summy KR, Escobar DE, Davis MR. An Overview of Aircraft Remote Sensing in Integrated Pest Management. Subtrop Plant Sci. 2003;55: 59–67. Available from: https://www.researchgate.net/publication/268049696_An_Overview_of_Aircraft_Remote_Sensing_in_Integrated_Pest_Management
5. Meagher RL, French JV. Augmentation of parasitoids for biological control of citrus blackfly in Southern Texas. Florida Entomol. 2004;87(2): 186–193. doi: 10.1653/0015-4040(2004)087[0186:AOPFBC]2.0.CO;2
6. Fletcher RS, Everitt JH, Davis MR, Escobar DE. Integrating Airborne Imagery and GIS Technology to Map and Compare Citrus Blackfly Infestations Occurring in Different Years. HORTTECH. 2004;14(3). doi: 10.21273/HORTTECH.14.3.0398
7. Smith HD, Maltby HL, Jimenez EJ. Biological control of the citrus blackfly in Mexico. U.S.D.A. Tech. Bull. 1964;1311. pp. 30.
8. Alvim RG, Aguiar-Menezes EL, Lima AF. Dissemination of *Aleurocanthus woglumi* in citrus plants, its natural enemies and new host plants in the state of Rio de Janeiro, Brazil. Ciência Rural. 2016;46(11): 1891–1897. doi: 10.1590/0103-8478cr20151101
9. Almeida MC de, Lhano MG. Ocorrência de *Aleurocanthus woglumi* Ashby, 1915 (Hemiptera: Aleyrodidae) no Estado do Rio de Janeiro. Rev Agro@mbiente On-line. 2014;9(1):424–427. doi: 10.5327/Z 1982-8470201400031897
10. Raga A, Felippe N, Imperato R. Population Dynamic of Citrus Blackfly, *Aleurocanthus woglumi* (Hemiptera: Aleyrodidae), in Tahiti Lime in the eastern of the State of São Paulo, Brazil. Annu Res Rev Biol. 2016;11(1): 1–7. doi: 10.9734/ARRB/2016/28668
11. Raga A, Basilli JFM, Soares DZ. Oviposition pattern of citrus blackfly *Aleurocanthus woglumi* (Hemiptera: Aleyrodidae) on citrus plants. Idesia. 2012;30(2): 111–114. doi: 10.4067/S0718-34292012000200014
12. Lima BG, Farias P, Ramos EM, Sales TM, Silva AG. Economic injury level of citrus black-fly in commercial 'Pera-Rio' orange area. Rev. Bras. Frutic. 2017;39(3): e-461. doi: 10.1590/0100-29452017461

13. Gonçalves da Silva A, Paulo PR, Silva Siqueira D, Boiça Junior AL. *Aleurocanthus woglumi* (Hemiptera: Aleyrodidae) geostatistics analysis in agroforestry and monoculture systems in Oriental Amazon. Rev Colomb Entomol. 2014;40(2): 213–224. Available from: http://www.scielo.org.co/pdf/rcen/v40n2/v40n2a14.pdf
14. Molina RO, Carvalho Nunes WM, Gil LG, Fonseca Rinaldi DAM, Filho JC, Carvalho RCZ. First report of citrus *Aleurocanthus woglumi* Ashby (Hemiptera: Aleyrodidae) in the State of Paraná, Brazil. Brazilian Arch Biol Technol. 2014;57(4): 472–475. doi: 10.1590/S1982-88372014000100003
15. Castilhos RV, Brugnara EC, Sabião RR, Andrade TPR, Negri G. First record of *Aleurocanthus woglumi* (Hemiptera: Aleyrodidae) in the state of Santa Catarina, Brazil (In Portuguese). 2019;40, doi: 10.4322/crt.18919
16. Gomes AMSV, Reis FO, Lemos RNS, Mondego JM, Braun H, Araujo JRG. Physiological characteristics of citrus plants infested with citrus blackfly. Rev Bras Entomol. 2019;63(2): 119–123. doi: 10.1016/j.rbe.2019.02.002
17. Medeiros FR, Lemos RNS, Ottati ALT, Araújo JRG, Machado KKG, Rodrigues AAC. Dinâmica populacional da mosca-negra-dos-citros *Aleurocanthus woglumi* Ashby (Hemiptera: Aleyrodidae) em *Citrus* spp. no Município de São Luís - MA. Rev Bras Frutic. 2009;31(4):1016–1021. doi: 10.1590/S0100-29452009000400014
18. Vieira DL, Ottati ALT, Lemos RNS, Lopes GS, Araujo JRG. Population fluctuation and spatial dependence of *Aleurocanthus woglumi* Ashby, 1915 (Hemiptera: Aleyrodidae) on *Citrus latifolia*. Rev Bras Frutic. 2014;36(4). doi: 10.1590/0100-2945-415/13
19. Correia RG, Cesar A, Lima S, Roberto P, Farias S, Clemilto F, et al. Primeiro registro da ocorrência de mosca-negra-dos-citros, *Aleurocanthus woglumi* Ashby, 1915 (Hemiptera: Aleyrodidae) em Roraima. 2011;245–248. doi: 10.18227/1982-8470ragro.v5i3.487
20. da Silva Gonçalves M. Flutuação Populacional Da Mosca-Negra-Dos-Citros, *Aleurocanthus woglumi* (Hemiptera: Aleyrodidae) e de seus inimigos naturais em um plantio de citros, Manaus, Amazonas, Brazil. Ministério Da Ciência E Tecnologia E Inovação-Mcti Instituto Nacional De Pesquisas Da Amazônia-Inpa Programa De Pós-Graduação Em Entomologia. 2013. Available from: https://bdtd.inpa.gov.br/bitstream/tede/1253/1/Maiara%20da%20Silva%20Goncalves.pdf
21. Lima BMFV, Almeida JEM de, Moreira JOT, Santos LC dos, Bittencourt MAL. Entomopathogenic fungi associated with citrus blackfly (*Aleurocanthus woglumi* Ashby) in Southern Bahia. Arq Inst Biol (Sao Paulo). 2018;84(0):1–4. doi: 10.1590/1808-1657000102015
22. Monteiro BS, Rodrigues KCV, Silva AG, Barros R. Ocorrência da Mosca-Negra-dos-Citros (*Aleurocanthus woglumi* Ashby) (Hemiptera: Aleyrodidae) em Pernambuco. Rev Caatinga. 2012;25(2): 173-176. Available from: https://periodicos.ufersa.edu.br/index.php/caatinga/article/view/2268/pdf
23. Silva JDC, Beserra-Junior JEA, Girão-Filho JE, Silva RBQ, Medeiros WR, Carvalho DS, et al. First report of citrus blackfly (Hemiptera: Aleyrodidae) in the state of Piauí, Brazil. Brazilian J Biol. 2015;75(2):499–500. doi: 10.1590/1519-6984.09214
24. Dietz HF, Zetek J. The blackfly of citrus and other subtropical plants. USDA Bulletin 1920;885: 1–55.
25. Bennet FD. Some recent successes in the field of biological control in the West Indies. Rev Per Entom. 1971;14(2):369–373.
26. Lopez VF, Kairo MTK, Pollard GV, Pierre C, Commodore N, Dominique D. Post-release survey to assess impact and potential host range expansion by *Amitus hesperidum* and *Encarsia perplexa*, two parasitoids introduced for the biological control of the citrus blackfly, *Aleurocanthus woglumi* in Dominica. BioControl. 2009;54(4): 497–503. doi: 10.1007/s10526-008-9207-4
27. White GL, Parkinson K. Field rearing of *Amitus hesperidum* silvestri (Hymenoptera: Platygasteridae) for control of citrus blackfly *Aleurocanthus woglumi* Ashby (Homoptera: Aleyrodidae) in Trinidad W.I. 37th Annual Meeting. July 15-20, 2001. Port of Spain, Trinidad and Tobago 256666, Caribbean Food Crops Society. Available from: https://ageconsearch.umn.edu/record/256666
28. Torres HAC. Biological control of the citrus blackfly (*Aleurocanthus woglumi* Ashby) by *Prospaltella opulenta* Silv. In central-western Venezuela: with a review of the pest's invasion of the Western Hemisphere and suppression by introduced parasites. 1930. Available from: https://archive.org/details/biologicalcontro00chav
29. Lopez SN. Primer registro de “la mosca negra de los cítricos” *Aleurocanthus woglumi* (Hemiptera: Aleyrodidae) en la Argentina First record of the citrus blackfly *Aleurocanthus woglumi* (Hemiptera: Aleyrodidae) in Argentina. Rev la Soc Entomológica Argentina. 2011;70(3–4):373–374. Available from: http://www.scielo.org.ar/pdf/rsea/v70n3-4/v70n3-4a23.pdf

30. Kajita H, Alam MZ. Whiteflies on guava and vegetables in Bangladesh and their aphelinid parasitoids. App. Entomol. Zool.1996;31(1):159-162. doi: 10.1303/aez.31.159
31. Aruna J, Jagginavar SB, Karabhantanal S. Seasonal incidence of citrus blackfly, *Aleurocanthus woglumi* Ashby and its natural enemies on acid lime. J. Exp. Zool. India. 2017;20: 1519–1523. doi: 10.20546/ijcmas.2019.805.046
32. Poovizhiraja B, Chinniah C, Murugan M, Irulandi S, Arutkani Aiyanathan KE, Balamohan TN. Population Dynamics and Seasonal Incidence of Major Sucking Pests of Acid Lime, *Citrus aurantifolia* Swingle. Int J Curr Microbiol Appl Sci. 2019;8(05):386–393. doi: 10.20546/ijcmas.2019.805.046
33. Chatterjee H. Distribution pattern of citrus blackfly (*Aleurocanthus woglumi* Ashby) infesting mandarin orange in Darjeeling district of West Bengal. Indian J. Agric. Res. 2002;36: 2133-2136.
34. Vasantharaj D. The Whitefly or Mealywing bugs: Bioecology, Host Specificity and Management. LAP LAMBERT Academic Publishing. 2012
35. Narayan BD. Mass multiplication and compatibility of *Aschersonia aleyrodis* against chemical pesticides. Department of Plant Pathology, Dr. Panjabrao Deshmukh Krishi Vidyapeeth, Akola. M. Sc. 2019; Print. xii, 76p. (Unpublished). Available from: https://krishikosh.egranth.ac.in/handle/1/5810146584
36. Available from: https://aicrp.icar.gov.in/fruits/achievements/protection-technology/ [accessed 16 October 2020]
37. Singh S, Reddy PVR, Deka S. Sucking Pests of Citrus. In: Sucking Pests of Crops (Omkar Ed.) Springer, Singapore. 2020. pp. 515. doi: 10.1007/978-981-15-6149-8
38. Pandey N, Singh A, Rana VS, Rajagopal R. Molecular characterization and analysis of bacterial diversity in *Aleurocanthus woglumi* (Hemiptera: Aleyrodidae). Environ Entomol. 2013;42(6): 1257–64. doi: 10.1603/EN13110
39. Susrama IGK, Supartha IW. Community structure of citrus leaf-sucking insect pest on citrus orchard in Gianyar regency, Bali province. Proceeding International Conference on Biosciences and Biotechnology 7th ICBB 2016 entitled “Biosciences and Biotechnology for a Sustainable Life”.
40. Samin N, Ghahari H, Behnood S. A contribution to the knowledge of whiteflies (Hemiptera: Aleyrodidae) in Khorasan and Semnan Provinces, Iran. Acta Phytopathol Entomol Hungarica. 2015;50(2): 287–295. doi: 10.1556/038.50.2015.2.12
41. Available from: https://qbank.eppo.int/arthropods/taxon/ALECWO/specimen/6956 [accessed 20 October 2020]
42. Kinawy MM, Al-Waili HM, Almandhari AM. Review of the successful classical biological control programs in Sultanate of Oman. Egypt. J. Biol. Pest Control. 2008;18(1): 1–10. Available from: https://www.cabi.org/isc/FullTextPDF/2009/20093037717.pdf
43. Tayyib M. Diversity of whiteflies (Aleurodidae: Homoptera) in Punjab (Pakistan). Doctor of Philosophy Inagri. Entomology Department of Agri. Entomology Faculty of Agriculture University of Agriculture Faisalabad Pakistan. 2013.
44. Bellis GA, Donaldson JF, Carver M, Hancock DL, Fletcher MJ. Records of Insect Pests on Christmas Island and the Cocos (Keeling) Islands, Indian Ocean. Aust Entomol. 2004;31(3): 93–102. Available from: https://search.informit.org/doi/10.3316/informit.069859139662723
45. Barro PJ, Liebregts W, Carver M. Distribution and identity of biotypes of *Bemisia tabaci* (Gennadius) (Hemiptera: Aleyrodidae) in member countries of the Secretariat of the Pacific Community. Australian Journal of Entomology. 1998;37(3): 214-218. doi: 10.1111/j.1440-6055.1998.tb01574.x
46. Van den Berg MA, Greenland J. Pest status of two blackfly species on citrus in South Africa and Swaziland. African Plant Prot. 2001;7(1): 53–57. Available from: https://journals.co.za/doi/abs/10.10520/EJC87827
47. Olubayo F, Kilalo D, Obukosia S, Shibairo S, Kasina M. Homopteran pests complex of citrus (*Citrus sinensis*) in semi-arid Kenya. Int. J. Sustain. Crop Prod. 2011;6(2): 23-28. Available from: https://profiles.uonbi.ac.ke/dchao/files/homoptera_pests_of_citrus.pdf
48. Hill D. Agricultural Insect Pests of the Tropics & their Control. Cambridge University Press. 1975. pp.576.
49. Oyelade OJ, Ayansola AA. Diversity and distribution of whiteflies in southwestern Nigeria. African Crop Science Journal. 2015;23: 135-149. Available from: https://www.ajol.info/index.php/acsj/article/view/117735

50. Bedford ECG, Thomas ED. Biological control of the Citrus Blackfly *Aleurocanthus woglumi* (Ashby) (Homoptera: Aleurodidae) in South Africa Journal Ent. Soc. S. Africa. 1965;28(1): 117-132. Available from: https://hdl.handle.net/10520/AJA00128789_3068
51. Singano Seguni Z, Mfugale O. Biological Control of Citrus Woolly White Fly *Aleurothrixus floccosus* Mask (Homoptera: Aleyrodidae) By Cales *noacki* How (Hymenoptera: Aphelinidae) in Some Tanzanian Small-Scale Citrus Orchards. Int J Res -GRANTHAALAYAH. 2016;4(6): 82–91. doi: 10.29121/granthaalayah.v4.i6.2016.2641

B) Known administrative areas (polygons; coordinates are for the polygon centroids).

Continent	Location	Latitude (DD)	Longitude (DD)	Reference number
North America	USA, Florida, Palm Beach	26.647500	-80.436389	1
North America	USA, Florida, Miami-Dade	25.608889	-80.498611	1
North America	USA, Florida, Manatee	27.477500	-82.357500	1
North America	USA, Florida, Polk	27.948611	-81.697500	1
North America	USA, Florida, Marion	29.210000	-82.056667	1
North America	USA, Florida, Volusia	29.063333	-81.148333	1
North America	USA, Florida, Alachua	29.674722	-82.357500	1
North America	USA, Hawaii, Honolulu, Oahu island	21.433333	-157.966667	2, 3
North America	USA, Hawaii, Hawaii, Big island	19.533481	-155.664722	2, 3
North America	USA, Hawaii, Maui island	20.867500	-156.616944	2, 3
North America	USA, Hawaii, Kauai island	22.049167	-159.530833	2, 3
North America	USA, Hawaii, Kalawao, Molokai island	21.133381	-157.012222	2, 3
Asia	China, Guangdong	23.500000	113.250000	4, 5
Asia	Iran, Fars	29.000000	53.000000	6
Asia	Iran, Khuzestan	31.500000	49.000000	7
Asia	Iran, Mazandaran	36.250000	52.333333	4, 8
Asia	Iran, Golestan	37.250000	55.000000	4, 6
Asia	Oman, Al-Batinah	23.765556	57.252500	9
Asia	Yemen, Lawdar & Mudiyah Abyan	13.628889	46.125833	10
Asia	Yemen, Lahij	13.166667	44.583333	11
Asia	Pakistan, Khyber Pakhtunkhwa	33.983056	71.686111	12

References

1. Nguyen R, Hamon AB, Fasulo TR. Citrus blackfly, *Aleurocanthus woglumi*. Gainesville Florida Department of Agriculture & Consumer Service - Division of Plant Industry. 1998; 3. EENY-42. Available from: https://edis.ifas.ufl.edu/pdf%5CIN%5CIN19900.pdf
2. Culliney TW, Nagamine WT. Introductions for Biological Control in Hawaii 1997–2001. Proc. Hawaiian Entomol. Soc. 2003; 36:145-153.
3. Heu, RA, Nagamine WT. Citrus blackfly, *Aleurocanthus woglumi* Ashby (Homoptera, Aleyrodidae). New Pest Advisory, 2001; 99-03. Available from: https://hdoa.hawaii.gov/pi/files/2013/01/npa99-03_citrusbf.pdf
4. Abd-Rabou S, Ghahari H, Myartseva SN, Ruíz-Cancino E. Iranian Aphelinidae (Hymenoptera: Chalcidoidea). J Entomol Zool Stud. 2013; 1(4):116–140. Available from: https://www.entomoljournal.com/archives/2013/vol1issue4/PartB/15.pdf
5. Clausen CP, Berry PA. The citrus blackfly in Asia and the importation of its natural enemies into tropical America. Technical Bulletin No 320. United States Department of Agriculture, Washington, D.C. 1932. pp. 58.
6. Abd-Rabou S, Ghahari H, Evans G, Iranian Eretmocerus-species including two new species (Hymenoptera: Chalcidoidea: Aphelinidae) Parasitoids of Whiteflies (Sternorrhyncha: Aleurodidae). Mitt. internat. entomol. 2005, 157-176.
7. Riley GB, Linkfield RL, Gardenhire RQ. Summary of Insect Conditions in Iran. In: Cooperative Economic Insect Report. Survey and Detection Operations. Plant Pest Control Division. Agricultural Research Service. United States Department of Agriculture. 1958;8(1): 134-136.
8. Ghahari H, Buhl NP. Check-list of Iranian Platygastridae (Hymenoptera, Platygastroidea). 2011;Band 32, Heft 22: 329-336.
9. Available from: http://5.162.223.177/Pages/AgrisAp_View.aspx?file=EN/OM/104042/0;10000;10007;10005;10003;10004;10011;10013;/OM2011007507.xml
10. Ba-Angood SAS. Field trials for the control of *Aleurocanthus woglumi* in the Yemen. Pans. 1977;23(2): 149–152. doi: 10.1080/09670877709412420
11. Citrus pest problems and their control in the Near East edited by Mors JG, Luck RF, Gumpf DJ. Rome: Food and Agriculture Organization of the United Nations, 1996 xi. pp. 403.
12. The West Pakistan Agricultural Pests Ordinance (W. P. Ordinance No. XXVIII of 1959). Available from: https://kpcode.kp.gov.pk/uploads/The_West_Pakistan_Agricultural_Pests_Ordinance_1959.pdf
