# Supplementary material for: Potential global distribution of Aleurocanthus woglumi considering climate change and irrigation
Source: PLoS One. 2021 Dec 20;16(12):e0261626. doi: 10.1371/journal.pone.0261626 (PMC8687537; doi:10.1371/journal.pone.0261626)
Supplement: S2 Table — (PDF) [file pone.0261626.s010.pdf]

S2 Table. CLIMEX parameter sensitivity values for *Aleurocanthus woglumi* parameters listed in Table 1, as applied to the CM30 1995H V2 global dataset under a natural rainfall scenario.

		Parameter Values			% Range	EI	% Core Distribution	Growth Variable Changes			Stress Variable Changes		
**Parameter**	**Mnemonic**	**Low**	**Default**	**High**	**Change**	**Change**	**Change**	**MI**	**TI**	**GI**	**CS**	**HS**	**DS**
Dry Stress Threshold	SMDS	0.0	0.1	0.2	5.3	10.4	9.4	0.0	0.0	0.0	0.0	0.0	70.1
Dry Stress Rate	HDS	-0.1	-0.1	0.0	0.9	1.7	0.1	0.0	0.0	0.0	0.0	0.0	7.7
Cold Stress Degree-day Threshold	DTCS	5.0	6.0	7.0	0.4	0.9	0.1	0.0	0.0	0.0	4.5	0.0	0.0
Limiting low moisture	SM0	0.0	0.1	0.2	0.4	2.5	0.0	19.0	0.0	4.3	0.0	0.0	0.0
Cold Stress Degree-day Rate	DHCS	0.0	0.0	0.0	0.3	0.7	0.0	0.0	0.0	0.0	3.4	0.0	0.0
Limiting low temperature	DV0	13.0	14.0	15.0	0.2	1.0	0.0	0.0	1.9	1.3	0.0	0.0	0.0
Degree-days per Generation	PDD	788.0	985.0	1182.0	0.2	0.6	0.0	0.0	0.0	0.0	0.0	0.0	0.0
Lower optimal moisture	SM1	0.2	0.3	0.4	0.1	2.1	0.0	15.3	0.0	3.1	0.0	0.0	0.0
Cold Stress Temperature Threshold	TTCS	-1.0	0.0	1.0	0.1	0.4	0.0	0.0	0.0	0.0	3.2	0.0	0.0
Lower optimal temperature	DV1	25.0	26.0	27.0	0.0	1.7	0.0	0.0	3.7	2.1	0.0	0.0	0.0
Cold Stress Temperature Rate	THCS	0.0	0.0	0.0	0.0	0.1	0.0	0.0	0.0	0.0	0.5	0.0	0.0
Upper optimal temperature	DV2	31.0	32.0	33.0	0.0	1.4	0.0	0.0	3.3	1.7	0.0	0.0	0.0
Limiting high moisture	SM3	1.9	2.0	2.1	0.0	2.0	0.0	2.4	0.0	2.1	0.0	0.0	0.0
Upper optimal moisture	SM2	1.1	1.2	1.3	0.0	2.9	0.0	4.5	0.0	3.1	0.0	0.0	0.0
Limiting high temperature	DV3	42.0	43.0	44.0	0.0	0.2	0.0	0.0	1.6	0.4	0.0	0.0	0.0
Heat Stress Temperature Threshold	TTHS	42.0	43.0	44.0	0.0	0.0	0.0	0.0	0.0	0.0	0.0	6.3	0.0
Heat Stress Temperature Rate	THHS	0.0	0.0	0.0	0.0	0.0	0.0	0.0	0.0	0.0	0.0	1.0	0.0
